# Feasibility and Preliminary Effectiveness of a Mobile App–Based Personalized Exercise Program in Older Patients With Chronic Knee Osteoarthritis: Pilot Randomized Controlled Trial

**DOI:** 10.2196/71073

**Published:** 2025-12-16

**Authors:** Yu Jin Im, Jihong Choi, Sol-a Choi, Jeong-Yi Kwon, Jong Geol Do

**Affiliations:** 1 Department of Physical and Rehabilitation Medicine, Samsung Medical Center, Sungkyunkwan University School of Medicine, 81 Irwon-ro, Gangnam-gu, Seoul, 06351, Republic of Korea, 82 02-3410-2811, 82 02-3414-2832

**Keywords:** osteoarthritis, mobile health, multimonitoring, pain, physical function, quality of life, mHealth, digital health

## Abstract

**Background:**

Knee osteoarthritis is a prevalent cause of disability among older adults, emphasizing the need for effective and accessible self-management strategies. Mobile app–based personalized exercise programs predominantly overcome the barriers associated with traditional approaches.

**Objective:**

This study aimed to evaluate the feasibility and preliminary efficacy of a 6-week mobile app–based self-exercise program that incorporates a multimonitoring system, weekly progress tracking, and dynamic exercise adjustments used by physiotherapists, and compares them with those of a conventional paper-based self-exercise program in older patients with chronic knee osteoarthritis.

**Methods:**

A total of 29 participants aged ≥60 years with chronic knee pain and radiographic evidence of osteoarthritis were randomized at a 2:1 ratio to either the intervention (19/29, 66%; mobile app–based program) or control (10/29, 34%; paper-based program) group. The mobile app delivered a personalized exercise program, which was tailored by physiotherapists based on remote monitoring of patient-reported symptoms. Feasibility outcomes included retention, adherence, and satisfaction rates, as well as safety. Preliminary clinical outcomes included changes from baseline to 6 weeks in the Western Ontario and McMaster Universities Osteoarthritis Index (WOMAC) total score, Numeric Rating Scale (NRS) pain, and other functional measures.

**Results:**

A total of 26 participants (n=16, 62% intervention and n=10, 38% control) completed the 6-week assessment, with retention rates of 84% and 100%, respectively. No adverse events were reported. Adherence was high in the intervention group, with 69% exercising ≥5 days per week and 88% reporting high satisfaction. The intervention group exhibited significant reductions in the WOMAC total score (median change –11.00, 95% CI –23.00 to −2.50; *P*=.01) and NRS pain score (mean change –2.12, 95% CI –3.13 to −1.11; *P*<.001).

**Conclusions:**

The mobile app–based personalized exercise program was feasible, safe, and well-accepted among older patients with knee osteoarthritis. High adherence and satisfaction support the practicality of this approach, and preliminary improvements in pain and function suggest potential clinical benefit. A larger, adequately powered trial is warranted to confirm the effectiveness of digital self-exercise interventions for knee osteoarthritis management.

## Introduction

Knee osteoarthritis is the most prevalent form of osteoarthritis and is one of the leading causes of disability among the older population [1]. According to the Global Burden of Disease Study 2020, the global age-standardized prevalence of knee osteoarthritis was 4307.4 cases per 100,000 population [2]. With increasing life expectancy and BMI, a 74.9% increase is expected in the number of patients with knee osteoarthritis by 2050, compared with 2020, affecting an estimated 642 million individuals globally [2]. The growing prevalence of knee osteoarthritis imposes a substantial burden on patients and health care systems, primarily due to chronic pain, functional limitation, and decreased quality of life [3]. These challenges highlight the need for effective, accessible, and sustainable management strategies to mitigate symptoms and maintain physical function in individuals with knee osteoarthritis.

Exercise interventions effectively reduce pain and improve function in patients with knee osteoarthritis [4-7]. Considering the chronic nature of knee osteoarthritis and its frequent association with comorbidities, such as cardiovascular disease and diabetes mellitus, nonpharmacologic strategies, including patient education and exercise, are crucial for long-term self-management of knee osteoarthritis [1]. The advancements in digital technology and the widespread adoption of mobile devices have expanded the delivery modes of exercise programs. Among these, mobile app–based self-exercise programs have emerged as cost-effective solutions for overcoming barriers such as limited access to health care facilities [8,9]. Furthermore, mobile apps offering video-guided exercises and remote monitoring systems may improve exercise adherence and the effectiveness of self-directed exercise [10].

Although several randomized controlled trials (RCTs) on web- or mobile-based exercise programs have been conducted for managing knee osteoarthritis [10-19], the number of studies on mobile apps that integrate regular patient-reported symptom assessment and dynamically adjust exercise programs based on remote monitoring is limited [10-19]. The mobile app used herein addresses this gap by incorporating patient-reported outcomes, including pain levels and the Knee Injury and Osteoarthritis Outcome Score–Physical Function Short Form (KOOS-PS) [20,21]. This multimonitoring system allows patients to track their progress and physiotherapists and physiatrists to review patients’ symptoms weekly. Thus, physiotherapists and physiatrists can modify self-exercise programs, providing personalized and app–based feedback for optimal exercise outcomes.

This pilot RCT primarily aimed to evaluate the feasibility of a 6-week mobile app–based self-exercise program incorporating a multimonitoring system in older patients with chronic knee osteoarthritis. The secondary aim was to assess preliminary trends in pain reduction, physical function, and muscle strength compared with a conventional paper-based self-exercise program, with the goal of informing the design of a future large-scale trial.

## Methods

### Trial Design

This prospective, parallel, 2-armed, single-blinded, single-center RCT was prospectively registered at ClinicalTrials.gov (NCT05197010) using CONSORT-EHEALTH (Consolidated Standards of Reporting Trials of Electronic and Mobile Health Applications and Online Telehealth) guidelines (Checklist 1). The registered protocol comprised 2 separate RCTs involving different patient populations—chronic low back pain and knee osteoarthritis—each using a tailored version of the mobile app. This study analyzed the RCT conducted in the knee osteoarthritis cohort.

### Participants

The study participants were recruited from the Department of Physical and Rehabilitation Medicine at Samsung Medical Center, Seoul, Republic of Korea between October 2021 and December 2021. Study information was posted on the hospital’s bulletin boards and provided to patients clinically diagnosed with knee osteoarthritis who visited the outpatient rehabilitation clinic. Individuals who expressed interest received detailed information about the study, and written informed consent was obtained prior to screening. Eligibility assessments were conducted only for participants who provided consent. Inclusion criteria were: (1) age ≥60 years; (2) knee pain persisting for ≥3 months; (3) overall knee pain severity ≥4 on 11-point Numeric Rating Scale (NRS); (4) score ≥23 points on the Korean version of the Montreal Cognitive Assessment [22], indicating ability to comprehend exercise program; and (5) radiographic evidence of osteoarthritis with Kellgren-Lawrence (K-L) grade ≥2 [23]. K-L grade was assessed by a musculoskeletal specialist (JGD) with over 10 years of clinical experience in musculoskeletal imaging. For patients with bilateral knee osteoarthritis, the most symptomatic knee—defined as the knee with greater pain as reported by the patient—was used for eligibility screening. The same knee was consistently evaluated at baseline and follow-up outcome assessments. Participants who met the following criteria were excluded: (1) a history of knee surgery, (2) a history of intra-articular or periarticular knee injections within the past 3 months, (3) a history of systemic inflammatory disease, (4) a history of polyneuropathy, (5) a history of hemorrhagic or ischemic cerebral infarction, (6) a history of heart failure or chronic obstructive pulmonary disease affecting walking or daily activities, and (7) other medical conditions unsuitable for self-directed home exercise (eg, severe anemia and uncontrolled diabetes mellitus).

Data on demographics, anthropometric, medical history, and social history were obtained from the participants prior to randomization.

### Randomization

Eligible participants were randomized to the intervention or control group in a 2:1 ratio using block randomization with a block size of 6. A computer-generated randomization list was prepared at the study outset and was securely concealed in opaque envelopes. The randomization process was conducted by an independent researcher who was not involved in the outcome assessments, ensuring blinding to the treatment allocation and randomization. To further minimize bias, statistical analyses were performed by an independent analyst who was not involved in the randomization process, participant allocation, or data collection. Although the study was single-blinded, with participants aware of the use of a mobile app, the study hypothesis was not disclosed to avoid potential bias in patient-reported outcomes.

### Intervention Group

**Figure 1.**
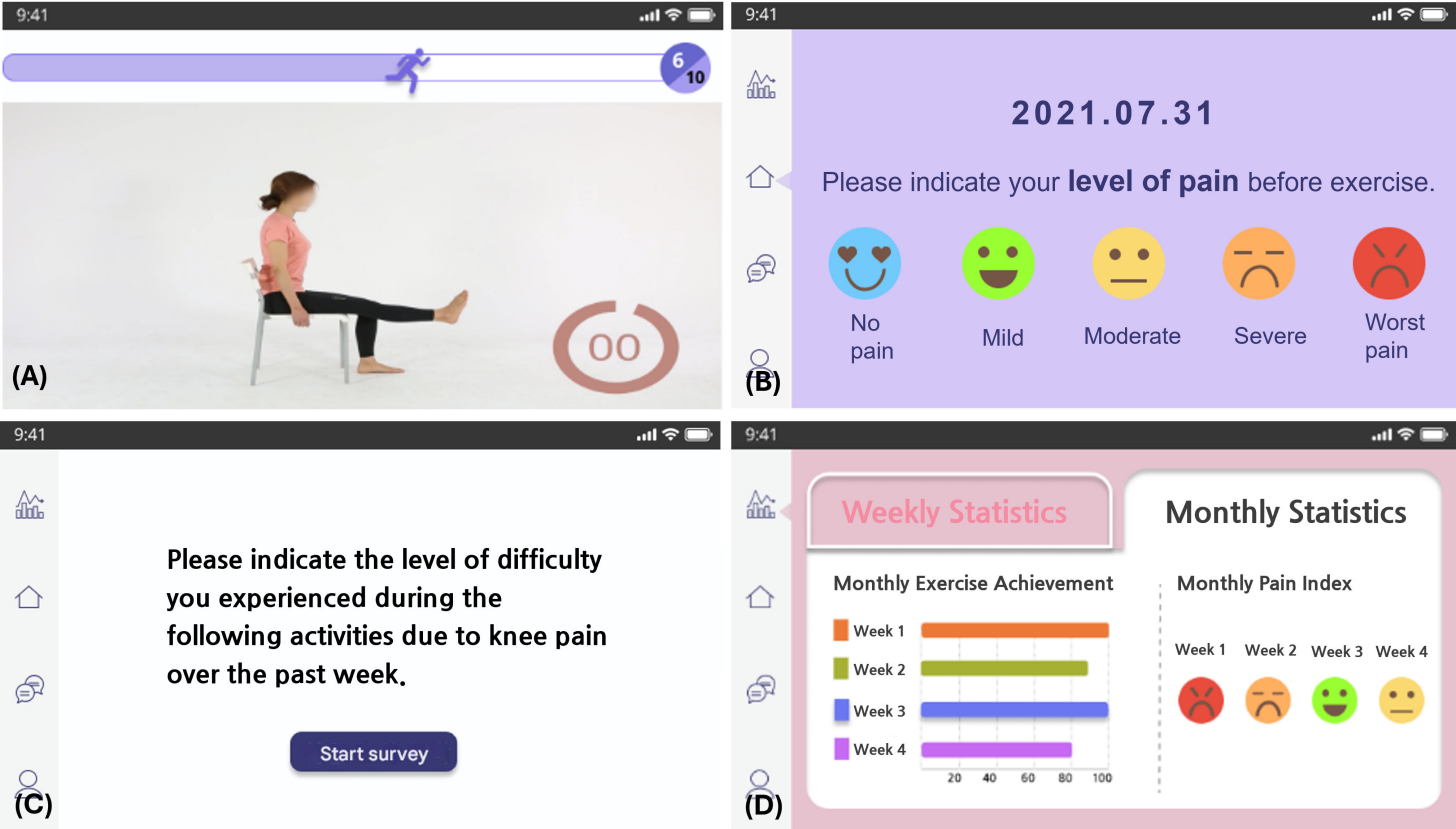
Features of the mobile application–based interactive exercise program (English translation of the original Korean version). The app is designed to facilitate self-managed knee rehabilitation via the following features: (A) instructional videos guiding users for performing prescribed exercises accurately within the app interface; (B) daily self-assessment of pain levels using a visual pain scale to monitor symptoms before exercise; (C) weekly tracking of self-reported outcomes, including functional difficulties due to knee pain, measured using the Knee Injury and Osteoarthritis Outcome Score–Physical Function short form; and (D) visualized statistics summarizing user progress, including weekly exercise achievement rates and pain indices, offering insights into rehabilitation progress over time.

**Figure 2.**
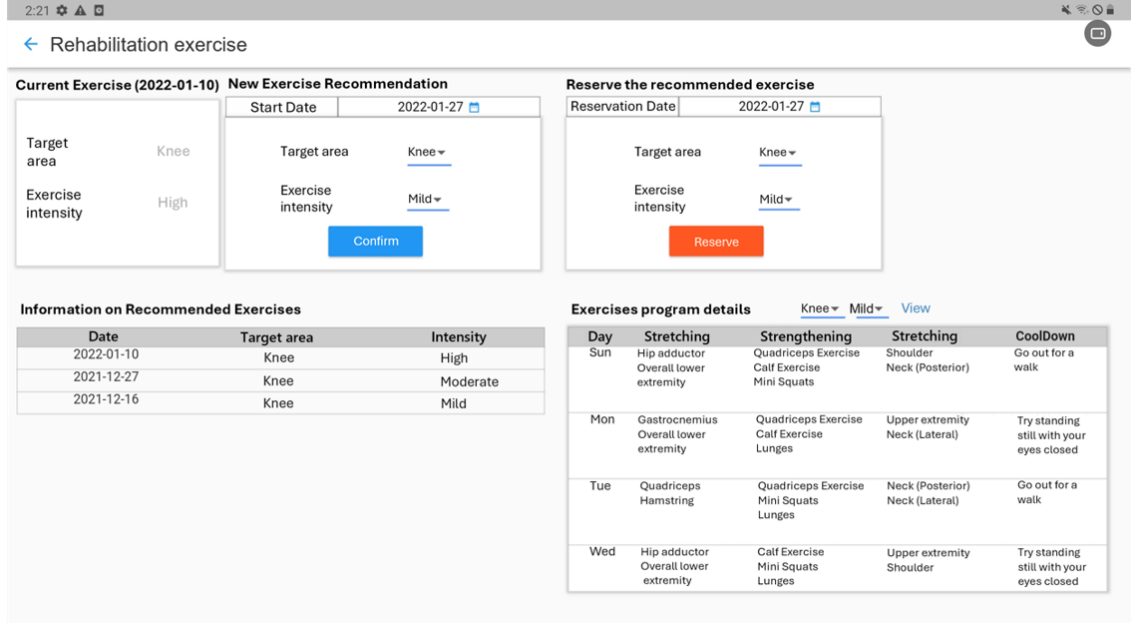
Exercise adjustment interface in mobile application for clinicians (English translation of the original Korean version). The app provides a platform for physical therapists and physiatrists to remotely monitor weekly patient-reported outcomes and assess the effectiveness of the current exercise program. Clinicians can review the patient’s ongoing exercise regimen and adjust the exercise intensity (mild, moderate, or high) accordingly.

The intervention group participants underwent a 6-week self-exercise program delivered via a mobile app–based self-exercise program (Multimedia Appendix 1). During their initial visit, participants were instructed to install the app on their mobile devices and received comprehensive training on its use. The app guided users through a daily 30-minute self-exercise protocol, comprising stretching, strengthening, functional, and cooldown exercises (Multimedia Appendix 1). The exercise program was tailored by physiotherapists based on the patient’s pain scale, functional status, and overall condition, ensuring a personalized approach to rehabilitation. To monitor the progress and adjust the intensity and content of the exercise program, the app incorporated a patient-reporting system. Before each daily exercise session, the participants quantified their pain intensity using a 5-point Likert scale. After completion, they again rated their perceived exertion using a 5-point Likert scale. On a weekly basis, the participants completed the KOOS-PS within the app. Accordingly, the app provided immediate visual feedback to the participants, and participants could check their weekly statistics for exercise achievement and pain levels within the app (Figure 1). Furthermore, these data were transmitted to the supervising physiotherapist and physiatrist for remote monitoring and exercise program modifications. The exercise program was adjusted weekly to 1 of the 3 intensity levels (ie, high, moderate, and mild) based on patient-reported outcomes (Figure 2). Owing to this approach, the app delivered a personalized self-exercise program throughout the 6-week intervention period.

### Control Group

Participants in the control group engaged in a 6-week self-exercise program, comprising daily 30-minute sessions without using a mobile app. During their initial visit, participants received a single session of individualized exercise education from a qualified physiotherapist. The exercise program components were the same as those in the intervention group, namely, stretching, strengthening and functional, and cooldown exercises. Furthermore, the participants were provided with a paper brochure detailing the prescribed exercise program.

No additional treatments for knee osteoarthritis, such as intra-articular injections, physical modalities, or surgical procedures, were initiated or prescribed during the 6-week intervention period in either group. Participants who were taking analgesics or nonsteroidal anti-inflammatory drugs for knee osteoarthritis at baseline were instructed to maintain their usual regimen without changes throughout the study period. Use of medications for other comorbid conditions was permitted. Any changes in additional treatments for knee osteoarthritis were monitored and documented at the 6-week follow-up visit.

### Primary Outcome: Feasibility Assessment

To evaluate feasibility, the retention rate was calculated as the proportion of participants who completed the 6-week follow-up assessment relative to those randomized. For participants who withdrew, the number and reasons for dropout were recorded. In addition, participants in the intervention group completed a survey to evaluate the mobile app–guided self-exercise program at the 6-week follow-up. The survey collected data on self-reported exercise frequency, participants’ expectations for achieving outcomes through mobile app use, and satisfaction levels with mobile app–guided self-exercise programs, as assessed using the North American Spine Society Patient Satisfaction Index [24]. For the safety outcome, any adverse events that occurred during the intervention period were recorded throughout the study.

### Secondary Outcome: Preliminary Efficacy Analysis

#### Overview

Exploratory comparisons of clinical outcomes were conducted to evaluate potential trends in treatment effect. The primary outcome of the preliminary efficacy analysis was the total score of the Western Ontario and McMaster Universities Osteoarthritis Index (WOMAC) [25,26]. The secondary outcomes included knee pain assessed using an NRS (11-point scale), KOOS-PS [20,21], Medical Outcomes Study 36-Item Short-Form Health Survey (SF-36) [27,28], Korean version of the Geriatric Depression Scale (K-GDS) [29,30], Timed Up-and-Go (TUG) test [31], and 30-second Chair Stand Test (30-CST) [32]. In addition, handgrip strength, knee extensor strength, and quadriceps femoris muscle thickness were included to provide a comprehensive evaluation of muscle strength and structure. Quadriceps muscle thickness was measured using ultrasonography. Although these variables (handgrip strength, knee extensor strength, KOOS-PS, and ultrasonographic assessment of quadriceps femoris muscle thickness) were not prespecified in the ClinicalTrials.gov registration (NCT05197010), they were incorporated prior to participant recruitment to enhance the assessment of physical function and musculoskeletal characteristics. The revised study protocol was approved by the institutional review board of Samsung Medical Center (2021-04-004). All outcomes were evaluated at baseline and at the 6-week follow-up. Baseline outcome data were collected from participants prior to randomization. Physical function tests were assessed by an experienced physical therapist (SAC), and ultrasound examinations were performed by an experienced physiatrist (JGD). Both assessors were blinded to group allocation throughout the study until completion of all data collection.

#### Western Ontario and McMaster Universities Osteoarthritis Index

The WOMAC was used to measure the physical function [25]. This index consisted of 24 items, including 5, 2, and 17 questions about pain, stiffness, and difficulties in daily activities (physical function), respectively. Each item was rated on a 5-point Likert scale ranging from 0=“none” to 4=“very severe”. The pain, stiffness, and difficulty in daily activities (physical function) subscales had score ranges of 0‐20, 0‐8, and 0‐68 points, respectively. Higher scores indicated greater physical dysfunction [25]. Herein, the Korean version of the WOMAC was used [26].

#### NRS Pain Severity

Subjective pain severity on the most symptomatic knee was assessed using the 11-point NRS. The total score for the scale used in this study ranged from 0 to 10, with 0=“no pain,” 5=“moderate pain,” and 10=“very severe pain.”

#### Knee Injury and Osteoarthritis Outcome Score–Physical Function Short Form

The KOOS-PS evaluated a patient’s ability to perform daily activities and physically demanding tasks [20,21]. It included 7 questions evaluating the subjective difficulty in various activities. Each item was scored on a scale of 0‐4, with a maximum total of 28 points. Higher scores indicated greater difficulty in performing daily life activities.

#### Medical Outcomes Study SF-36 Health Survey

The health-related quality of life was assessed using the SF-36 [27,28], which is a validated instrument comprising 36 items that evaluate 8 health domains [28]. Raw scores from each domain were summed and transformed into a standardized total score of 0‐100, with high scores indicating good health status.

#### Korean Version of the Geriatric Depression Scale

Depressive symptoms were assessed using the Geriatric Depression Scale, which consisted of 30 items, with high scores indicating severe depression levels [29]. Herein, K-GDS, adapted and standardized by Jung et al [30], was used.

#### TUG Test

The TUG test was performed per the Podsiadlo and Richardson protocol [31]. The test was conducted using a chair with armrests placed on a flat surface, with a marker located 3 meters away. The participants were allowed to use walking aids (eg, canes and walkers) if necessary; however, no physical assistance was provided by the guardians. The time taken to complete the task was recorded for 3 trials, and the average time was calculated and used for analysis.

#### 30-Second Chair Stand Test

The 30-CST was used to evaluate functional lower extremity strength by recording the total number of sit-to-stand repetitions a participant can complete within 30 seconds [32]. A low repetition count reflected reduced functional strength of the lower extremity muscles [32]. During the test, the participants were seated in chairs without armrests. If the participant could not perform the task or needed their arms for assistance, the test was terminated, and the score was marked as zero.

#### Handgrip Strength Test

The maximum handgrip strength of the dominant hand was measured using a MicroFET HandGRIP dynamometer (Hoggan Scientific) in the seated position. The highest value among the 3 consecutive trials was used for analysis.

#### Knee Extension Strength Test

The peak isometric force of the knee extensor muscles of the most symptomatic knee was assessed using a MicroFET2 Digital Handheld Muscle Tester (Hoggan Scientific). The participants were seated in a chair with their hands resting on their thighs. The device was positioned on the lower leg, resisting the direction of the participant’s movement. Two trials were performed, and the higher value was recorded for analysis.

#### Quadriceps Muscle Thickness

The thickness of the femoral quadriceps muscle was assessed bilaterally using a portable ultrasound device (Vscan Extend; GE Healthcare). An experienced physiatrist (JGD) with 12 years of experience in musculoskeletal ultrasonography performed ultrasonographic measurements using a linear probe. The participants were placed in the supine position with their knees completely extended. Muscle thickness was measured at the mid-thigh level, defined as the midpoint between the anterior superior iliac spine and the superior pole of the patella [33]. The probe was placed perpendicular to the femur’s long axis to acquire a transverse image of the rectus femoris and vastus intermedius muscles. Measurements were taken for both legs, recording the thickness of the rectus femoris and vastus intermedius, separately. At the 6-week follow-up, the thickness of the femoral quadriceps was remeasured at the same anatomical location using identical machine settings, including frequency and gain, to ensure consistency.

### Sample Size

According to the registered protocol of the main study (NCT05197010), a total of 60 participants (30 with chronic low back pain and 30 with knee osteoarthritis) were planned for enrollment. The sample size for each population was calculated based on recommendations from the literature on conducting pilot studies [34-36]. Assuming an expected effect size of 0.5 [6] and aiming for 90% power at a 2-sided significance level of 0.05, a total of 28 to 30 participants (14‐15 per group) were necessary for the pilot trial [35,36]. To facilitate recruitment and encourage participation, a 2:1 allocation ratio was applied, resulting in 20 and 10 participants in the intervention and control groups, respectively. This paper reports data from the knee osteoarthritis cohort only, which was one of the 2 independent randomized controlled trials (chronic low back pain and knee osteoarthritis) included in the registered main protocol (NCT05197010).

### Statistical Analysis

All analyses were performed using R (version 4.2.1; R Foundation for Statistical Computing). The primary analysis followed a modified intention-to-treat principle, including all randomized participants who received at least 1 session of the allocated intervention and had postbaseline outcome data available. This approach was adopted because excluding participants who did not initiate the intervention enables estimation of the treatment effect within the subpopulation who would have initiated treatment [37]. The Shapiro-Wilk test was used to assess data normality. Between-group comparisons of baseline characteristics and outcome measures at baseline and at the 6-week follow-up were performed using the Fisher exact test for categorical variables. For continuous variables, either the 2-tailed independent *t* tests or the Wilcoxon rank-sum test was used, based on the normality of the data.

The preliminary efficacy analysis evaluated the between-group differences in changes from baseline to 6-week follow-up. Between-group differences in changes (calculated as intervention group minus control group) were analyzed using independent *t* tests with mean differences and 95% CI when normality assumptions were met. Otherwise, the Wilcoxon rank-sum test was performed, and the median differences with 95% CI were estimated using the Hodges-Lehmann method. For within-group comparisons (calculated as 6-week follow-up minus baseline), a paired *t* test or a Wilcoxon signed-rank test was performed based on the normality of the data.

Statistical significance was set at *P*<.05 (2-tailed), and cases with missing data were excluded from the analysis.

### Ethical Considerations

The study protocol was reviewed and approved by the Institutional Review Board of Samsung Medical Center (2021-04-004) and was conducted in accordance with the Declaration of Helsinki. Written informed consent was obtained from all participants prior to enrollment. To ensure privacy and confidentiality, all collected data were deidentified. Participants received compensation for transportation costs of 50,000 KRW (approximately US $40) per visit, totaling 100,000 KRW (approximately US $80) for two visits, based on the exchange rate at the time of the study.

## Results

### Study Participants and Feasibility Outcomes

**Figure 3.**
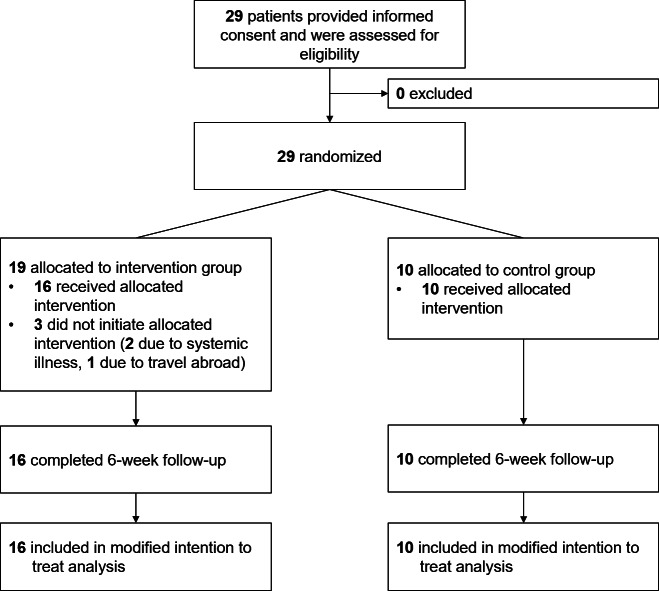
Study flowchart.

During the 3-month recruitment period, a total of 29 participants provided informed consent and underwent an eligibility assessment, and none met the exclusion criteria. Specific reasons for declining participation among individuals who refused screening were not recorded. Therefore, the participant flow diagram begins with the number of participants who provided consent and were screened (Figure 3). In this study, 29 participants (mean age 70.4, SD 6.6 y; n=24, 83% female participants) were randomized in a 2:1 ratio to the intervention and control groups. Three participants in the intervention group withdrew before initiating the assigned intervention. Two withdrew due to poor general condition related to systemic illness, and one withdrew because of travel abroad (Figure 3). These 3 participants were excluded from subsequent analyses. A total of 26 participants (n=16, 62% in the intervention group and n=10, 38% in the control group) completed the 6-week follow-up, yielding an overall retention rate of 90% (26/29; 16/19, 84% for the intervention group and 10/10, 100% for the control group). No adverse events were reported in either group during the study period. At baseline, there were no significant differences between the 2 groups in any clinical or demographic characteristics, including K-L grade (Tables1  2).

**Table 1.** Baseline characteristics of the participants (N=29).

Characteristics	Total	Intervention group (n=19)	Control group (n=10)
Age (y), mean (SD)	70.4 (6.6)	69.9 (6.8)	71.4 (6.4)
Sex, n (%)			
Male	5 (17)	3 (16)	2 (20)
Female	24 (83)	16 (84)	8 (80)
Height (cm), mean (SD)	157.4 (7.4)	157.8 (7.8)	156.7 (6.9)
Weight (kg), mean (SD)	63.6 (10.6)	62.7 (10.5)	65.2 (11.2)
BMI (kg/m^2^), mean (SD)	25.6 (3.6)	25.1 (3.6)	26.5 (3.5)
Radiographic score, n (%)^a^			
2	15 (52)	12 (63)	3 (30)
3	8 (28)	3 (16)	5 (50)
4	6 (21)	4 (21)	2 (20)
Single-person household, n (%)			
Yes	4 (14)	2 (11)	2 (20)
No	25 (86)	17 (89)	8 (80)
Visual impairment, n (%)			
Yes	0 (0)	0 (0)	0 (0)
No	29 (100)	19 (100)	10 (100)
Hearing impairment, n (%)			
Yes	0 (0)	0 (0)	0 (0)
No	29 (100)	19 (100)	10 (100)
Fall event in the past 1 year, n (%)			
Yes	0 (0)	0 (0)	0 (0)
No	29 (100)	19 (100)	10 (100)

a Kellgren-Lawrence grade.

**Table 2.** Outcome measures according to group at baseline.

Outcome measure	Baseline (0 wk), mean (SD)		
	Intervention group (n=19)	Control group (n=10)	*P* value
Primary outcome			
WOMAC^a^			
Total	34.16 (17.43)	35.50 (19.43)	.85
Pain	5.37 (3.35)	7.90 (4.89)	.16
Stiffness	3.21 (2.12)	2.90 (1.52)	.69
Physical function	25.58 (13.25)	24.70 (14.35)	.87
Secondary outcome			
NRS^b^ pain	5.58 (1.89)	5.60 (1.35)	.63
KOOS-PS^c^	12.11 (5.57)	14.30 (7.97)	.39
SF-36^d^	73.21 (17.92)	68.50 (20.44)	.53
K-GDS^e^	10.00 (6.92)	10.80 (6.55)	.77
TUG^f^ (s)			
Right	8.41 (2.92)	8.73 (1.85)	.75
Left	9.09 (2.50)	8.78 (2.13)	.74
30-second Chair Stand (n^g^)	12.32 (3.76)	13.50 (2.92)	.39
Grip strength (kgf)	20.43 (7.75)	21.23 (7.19)	.63
Knee extensor strength (kgf)	20.80 (6.12)	21.75 (5.27)	.33
Muscle thickness (cm)			
Right RF^h^	0.94 (0.25)	0.91 (0.14)	.77
Left RF	0.93 (0.27)	0.92 (0.23)	.93
Right VI^i^	0.99 (0.34)	1.05 (0.36)	.69
Left VI	1.03 (0.31)	1.15 (0.28)	.33

a WOMAC: Western Ontario and McMaster Universities Osteoarthritis Index.

b NRS: Numerical Rating Scale.

c KOOS–PS: Knee Injury and Osteoarthritis Outcome Score–Physical Function Short Form.

d SF-36: 36-Item Short-Form Health Survey.

e K-GDS: Korean version of the Geriatric Depression Scale.

f TUG: Timed Up-and-Go test.

g the number of chair-stand repetitions completed during the 30-second chair stand test.

h RF: rectus femoris.

i VI: vastus intermedius.

All 16 participants in the intervention group completed the mobile app–related survey. During the intervention period, adherence to the mobile app–based exercise program was high: 69% (n=11) of participants reported exercising ≥5 days per week, and 38% (n=6) reported daily exercise. The remaining participants reported exercising at least twice weekly, with 13% (n=2) exercising 2 to 3 days per week and 19% (n=3) exercising 3 to 5 days per week.

Regarding participants’ goals with our mobile app, 50% (n=8) of participants aimed for pain relief, 44% (n=7) sought improvement in muscle strength, and 6% (n=1) expected enhanced quality of life. Overall, 88% (n=14) reported high satisfaction (scores 1‐2 on the North American Spine Society Patient Satisfaction Index). None of the participants in either group changed their medication regimen or received additional treatments for knee osteoarthritis during the study period.

### Preliminary Efficacy Analysis

The WOMAC total score exhibited a significantly greater reduction in the intervention group compared with the control group from baseline to the 6-week follow-up (between-group median difference in change –12.00, 95% CI –28.00 to –2.00; *P*=.02), indicating a significantly greater improvement in the intervention group (Tables3  4). Within-group analysis revealed a significant reduction in the WOMAC total score in the intervention group (median change –11.00, 95% CI –23.00 to −2.50; *P*=.01), whereas no significant changes were observed in the control group (median change 4.45, 95% CI –16.00 to 20.00; *P*=.39; Table 5).

**Table 3.** Outcome measures according to group at 6-week follow-up.

Outcome measure	Follow-up (6 wk), mean (SD)		
	Intervention group (n=16)	Control group (n=10)	*P* value
Primary outcome			
WOMAC^a^			
Total	23.50 (17.66)	38.50 (20.23)	.06
Pain	4.69 (3.50)	8.40 (3.20)	.01
Stiffness	2.00 (1.46)	2.70 (2.11)	.41
Physical function	16.81 (13.11)	27.30 (16.36)	.08
Secondary outcome			
NRS^b^ pain	3.12 (2.00)	5.50 (2.22)	.01
KOOS–PS^c^	9.94 (7.42)	17.20 (10.75)	.05
SF-36^d^	82.19 (22.45)	72.40 (22.09)	.33
K-GDS^e^	10.12 (8.11)	10.90 (6.69)	.44
TUG^f^ (s)			
Right	7.79 (1.38)	8.63 (1.39)	.14
Left	7.92 (1.63)	8.91 (2.06)	.19
30-second Chair Stand (n^g^)	17.00 (15.03)	14.40 (4.70)	.79
Grip strength (kgf)	22.26 (8.15)	22.06 (8.61)	.99
Knee extensor strength (kgf)	27.24 (7.88)	27.87 (6.23)	.53
Muscle thickness (cm)			
Right RF^h^	0.87 (0.24)	0.85 (0.26)	.85
Left RF	0.94 (0.29)	0.88 (0.16)	.49
Right VI^i^	1.16 (0.42)	1.05 (0.37)	.51
Left VI	1.01 (0.33)	1.19 (0.29)	.16

a WOMAC: Western Ontario and McMaster Universities Osteoarthritis Index.

b NRS: Numerical Rating Scale.

c KOOS-PS: Knee Injury and Osteoarthritis Outcome Score–Physical Function Short Form.

d SF-36: 36-Item Short-Form Health Survey.

e K-GDS: Korean version of the Geriatric Depression Scale.

f TUG: Timed Up-and-Go test.

g “n” refers to the number of chair-stand repetitions completed during the 30-second chair stand test.

h RF: rectus femoris.

i VI: vastus intermedius.

**Table 4.** Between-group differences in changes in outcome measures from baseline to 6 weeks.

Outcome measure	Difference in change between groups (intervention group vs control group)	
	Mean difference (95% CI)	*P* value
Primary outcome		
WOMAC^a^		
Total^b^	–12.00 (–28.00 to –2.00)	.02
Pain	–1.50 (–5.22 to 2.22)	.40
Stiffness	–0.99 (–2.75 to 0.77)	.25
Physical function^b^	–10.00 (–25.00 to –4.00)	.01
Secondary outcome		
NRS^c^ pain	–2.02 (–3.94 to –0.11)	.04
KOOS-PS^b^^,^^d^	–4.00 (–7.00 to 1.00)	.14
SF-36^b^^,^^e^	7.00 (–9.00 to 22.00)	.41
K-GDS^f^	0.52 (–3.20 to 4.25)	.77
TUG^g^ (s)		
Right^b^	–1.01 (–2.57 to 0.14)	.07
Left	–0.57 (–1.89 to 0.75)	.38
30-second Chair Stand (n^h^)^b^	0.27 (–2.00 to 3.00)	.71
Grip strength (kgf)	0.54 (–2.36 to 3.44)	.71
Knee extensor strength (kgf)	0.62 (–4.14 to 5.39)	.79
Muscle thickness (cm)		
Right RF^i^	0.09 (–0.12 to 0.30)	.35
Left RF	0.00 (–0.23 to 0.24)	.98
Right VI^b^^,^^j^	0.13 (–0.12 to 0.31)	.32
Left VI^b^	0.17 (–0.23 to 0.46)	.28

a WOMAC: Western Ontario and McMaster Universities Osteoarthritis Index.

b Data did not meet the normality assumption. The Wilcoxon signed-rank test was used for intra-group analysis, and the Wilcoxon rank-sum test was employed used for between-group analysis. Median values (Hodges-Lehmann estimate of the median difference) with 95% confidence intervals (CI) are presented in this table for these outcomes.

c NRS: Numerical Rating Scale.

d KOOS-PS: Knee Injury and Osteoarthritis Outcome Score–Physical Function Short Form.

e SF-36: 36-Item Short-Form Health Survey.

f K-GDS: Korean version of the Geriatric Depression Scale.

g TUG: Timed Up-and-Go test.

h the number of chair-stand repetitions completed during the 30-second chair stand test.

i RF: rectus femoris.

j VI: vastus intermedius.

**Table 5.** Within-group changes in outcome measures from baseline to 6 weeks.

Outcome measure	Changes within groups (6 wk follow-up to baseline), mean change (95% CI)			
	Intervention group (n=16)	*P* value	Control group (n=10)	*P* value
Primary outcome				
WOMAC^a^				
Total^b^	–11.00 (–23.00 to –2.50)	.01	4.45 (–16.00 to 20.00)	.39
Pain	–1.00 (–2.72 to 0.72)	.23	0.50 (–2.98 to 3.98)	.75
Stiffness	–1.19 (–2.16 to –0.21)	.02	–0.20 (–1.77 to 1.37)	.78
Physical function^b^	–10.50 (–19.50 to –2.00)	.01	6.00 (–11.00 to 14.5)	.36
Secondary outcome				
NRS^c^ pain	–2.12 (–3.13 to –1.11)	*<*.001	–0.10 (–1.84 to 1.63)	.90
KOOS–PS^b^^,^^d^	–2.50 (–4.00 to 0.00)	.046	1.50 (–4.00 to 12.00)	.54
SF-36^b^^,^^f^	7.07 (–1.50 to 18.00)	.09	0.00 (–12.00 to 23.00)	.99
K-GDS^g^	0.62 (–1.57 to 2.82)	.55	0.10 (–3.16 to 3.36)	.95
TUG^h^ (s)				
Right^b^	–1.10 (–2.08 to –0.47)	.006	–0.11 (–0.85 to 1.50)	.88
Left	–0.65 (–1.79 to 0.50)	.25	–0.08 (–0.86 to 0.70)	.83
30-second chair stand (n^e^)^b^	1.50 (0.00 to 3.50)	.08	1.19 (–2.00 to 4.00)	.36
Grip strength (kgf)	1.41 (–0.97 to 3.79)	.23	0.87 (–1.06 to 2.80)	.33
Knee extensor strength (kgf)	6.74 (3.33 to 10.15)	*<*.001	6.12 (2.38 to 9.86)	.005
Muscle thickness (cm)				
Right RF^i^	0.05 (–0.05 to 0.14)	.30	–0.05 (–0.24 to 0.15)	.61
Left RF	–0.06 (–0.18 to 0.07)	.35	–0.06 (–0.27 to 0.16)	.56
Right VI^b^^,^^j^	0.04 (–0.05 to 0.17)	.26	–0.03 (–0.26 to 0.18)	.62
Left VI^b^	0.17 (0.02 to 0.27)	.03	–0.02 (–0.31 to 0.29)	.92

a WOMAC: Western Ontario and McMaster Universities Osteoarthritis Index.

b Data did not meet the normality assumption. The Wilcoxon signed-rank test was used for intragroup analysis, and the Wilcoxon rank-sum test was used for between-group analysis. Median values (Hodges-Lehmann estimate of the median difference) with 95% CI are presented in this table for these outcomes.

c NRS: Numerical Rating Scale.

d KOOS-PS: Knee injury and Osteoarthritis Outcome Score–Physical Function Short Form.

e the number of chair-stand repetitions completed during the 30-second chair stand test.

f SF-36: 36-Item Short-Form Health Survey.

g K-GDS: Korean version of the Geriatric Depression Scale.

h TUG: Timed Up-and-Go test.

i RF: rectus femoris.

j VI: vastus intermedius.

Among the WOMAC subscales, the physical function score exhibited significantly greater improvement in the intervention group than in the control group (between-group median difference in change –10.00, 95% CI –25.00 to –4.00; *P*=.01; Table 4). Within-group analysis revealed significant reductions in the stiffness and physical function scores in the intervention group (mean change in stiffness –1.19, 95% CI –2.16 to –0.21; *P*=.02; median change in physical function –10.50, 95% CI –19.50 to –2.00; *P*=.01), whereas no significant changes were observed in the control group (*P*>.05; Table 5).

A between-group comparison of changes from baseline to the 6-week follow-up revealed that the improvements in NRS pain were statistically greater in the intervention group than in the control group (between-group mean difference in change –2.02, 95% CI –3.94 to –0.11; *P*=.04). No significant differences in changes in the remaining secondary clinical outcome measures were observed between the 2 groups, including KOOS-PS, SF-36, K-GDS, TUG test, 30-CST, handgrip strength, knee extensor strength, and thickness of the quadriceps femoris muscle (*P*>.05 for all; Tables3  4).

Significant within-group improvements were observed in the intervention group at the 6-week follow-up, including reductions in NRS pain (mean change –2.12, 95% CI –3.13 to –1.11; *P*<.001), KOOS-PS (median change –2.50, 95% CI –4.00 to 0.00; *P*=.046), and TUG test for the right side (median change –1.10 s, 95% CI –2.08 to –0.47; *P*=.006). Furthermore, significant increases were noted in knee extensor strength (mean change 6.74, 95% CI 3.33-10.15; *P*<.001) and left vastus intermedius thickness (median change 0.17 cm, 95% CI 0.02-0.27; *P*=.03; Table 5). However, the control group exhibited no significant improvements in secondary outcomes, except for the knee extensor strength, which increased by 6.12 kgf (95% CI 2.38-9.86; *P*=.005).

## Discussion

### Principal Findings

This pilot randomized controlled trial demonstrated that a mobile app–based personalized self-exercise program was feasible, acceptable, and safe for older patients with knee osteoarthritis. The study achieved a high retention rate in both groups (16/19, 84% for the intervention group and 10/10, 100% for the control group). Although 3 participants in the intervention group withdrew, all reasons for withdrawal were unrelated to the mobile app or study procedures. These findings indicate good participant compliance and minimal burden over the 6-week intervention period. High adherence (nearly 70% performing exercise ≥5 d/wk) and strong participant satisfaction (14/16, 88% reporting high satisfaction) of the intervention group indicate that the program’s design—featuring personalized exercise adjustment and visualized feedback—was well tolerated and engaging for older users.

Although this pilot study was not powered to detect definitive efficacy outcomes, exploratory analyses revealed improvements in pain and physical function among participants using the mobile app compared with traditional paper-based methods. The observed effect sizes for physical function (WOMAC total: –0.63, 95% CI –1.11 to –0.15) and pain severity (NRS: −1.10, 95% CI –1.74 to –0.46) indicate the potential effectiveness of our mobile app. Furthermore, these clinical outcomes aligned with the patients’ needs, with 94% of the participants in the intervention group anticipating improvements in pain or muscle strength. Taken together, the favorable feasibility outcomes and preliminary clinical trends support the practicality and promise of conducting a larger, adequately powered randomized controlled trial to verify clinical effectiveness.

### Comparison With Prior Studies

A 2024 Cochrane systematic review reported that exercise interventions improve clinical outcomes, including immediate pain relief, physical function, and quality of life (absolute effect sizes: 0.72 for physical function; 0.71 for pain), compared with no treatment, usual care, or limited education [7]. Despite the established efficacy of exercise interventions for knee osteoarthritis, the limited use of digital delivery methods in these trials highlights a crucial gap in clinical studies. Only a small fraction (6%) of the reviewed trials incorporated digital delivery methods for exercise interventions, with most relying on in-person methods [7].

Reportedly, digital tools incorporating personalized feedback and monitoring improve pain management and functional outcomes in chronic musculoskeletal conditions [38-40]. For knee osteoarthritis, several studies have explored the use of mobile apps for home-based exercise or self-management in knee osteoarthritis [10,13-15,17-19]. Although 1 study used a decision-tree algorithm to determine the disease stage and recommend an appropriate exercise program for knee osteoarthritis at baseline [17] and some mobile apps tracked metrics such as exercise duration [10] and step counts [13], none monitored patient symptoms within the app consistently. The mobile app used in this study addressed this gap by combining the ongoing patient-reported symptom monitoring with responsive feedback and exercise modifications, representing a distinct advancement over existing mobile app interventions.

In a prior study using a paper-based home exercise program for knee osteoarthritis, the mean adherence rate at 6 weeks was 60.2% (SD 33.1%) [10]. In our study, 69% (11/16) of participants in the intervention group exercised 5 or more days per week, indicating a potential for improved exercise adherence with the use of a mobile app. By combining established exercise benefits with user-friendly technology, this mobile approach may enhance adherence and engagement, thereby supporting improved pain control, physical function, and overall quality of life in older patients with knee osteoarthritis.

### Strengths of This Study

A key strength of our app is the high retention and adherence rate, which is particularly significant considering the documented challenges in older populations [41]. The 2024 Cochrane systematic review noted a decline in the sustained efficacy of exercise at 12 months (absolute effect sizes reduced to 0.32 for pain and physical function) [7]. These findings highlight the importance of adherence in maintaining the long-term benefits of exercise interventions. The success of the app in maintaining adherence can be attributed to three key features. First, the automated pop-up alarms delivered via participants’ mobile phones are active reminders to encourage consistent participation in the exercise program. Second, the visualized statistical tracking features and weekly patient-reported symptom monitoring system of the app enable participants to continuously assess their progress, providing tangible feedback on their exercise achievements. Third, the dynamic modification of exercise intensity based on individual progression would maintain appropriate challenge levels while accommodating participants’ changing capabilities. These interactive features of digital technology may address common barriers to exercise adherence, as demonstrated by high participation rates and significant improvements in subjective and objective outcomes in the intervention group. Herein, the superior outcomes observed in participants who completed the mobile app–based self-exercise program compared with those who performed paper-based self-exercises may be attributed to the dynamic adjustments and real-time feedback of the program; these findings suggest that a user-friendly, personalized mobile app–based self-exercise program may achieve therapeutic benefits superior to traditional brochure-guided self-exercises and comparable to in-person interventions while enhancing accessibility and convenience for patients.

In addition to the clinical outcomes, this study identified possible physiological changes associated with the mobile app–based exercise program. In particular, an increase in the left vastus intermedius muscle thickness (median change 0.17 cm, 95% CI 0.02 to 0.27; *P*=.03) of the intervention group may indicate a trend toward muscle mass gain following participation in the program. This finding is noteworthy because reduced lower limb muscle strength and muscle mass are well-documented in symptomatic knee osteoarthritis [42] and risk factors for falls among older populations [43]. Patients with knee osteoarthritis are particularly susceptible to recurrent falls [44], and fall-related injuries are a leading cause of morbidity and mortality among the older population [41,45]. Thus, exercise interventions targeting muscle strength and mass are crucial for mitigating fall risk. In our study, within-group analysis revealed significant increases in knee extensor strength in the intervention and control groups. However, improvements in the TUG test, a key indicator of fall risk, were exclusively observed in the intervention group (median change –1.10 s, 95% CI –2.08 to −0.47; *P*=.006). This discrepancy may be attributed to the concurrent increase in the vastus intermedius muscle mass observed in the intervention group, underscoring the potential of mobile app–based self-exercise programs in reducing the risk of falls in patients with knee osteoarthritis.

Nevertheless, this study was preliminary, and ultrasound-based muscle thickness measurements are subject to measurement variability [46]. The increase observed only in the left vastus intermedius may represent measurement variation or asymmetrical limb use rather than a consistent hypertrophic response of the muscle. Therefore, these findings should be interpreted as exploratory, and confirmation in a larger, well-controlled trial is warranted.

### Limitations

The relatively small sample size and the high proportion of female participants in the study sample (24/29, 83%) may limit the generalizability of the findings. Another limitation of this study is that exercise adherence was assessed only in the mobile app group, and direct comparisons with the paper-based control group were not conducted. Therefore, it remains unclear whether the higher adherence observed reflects the effect of the digital intervention itself or general participant motivation. In addition, the relatively short 6-week follow-up period may be insufficient to completely assess the sustainability of the observed improvements. Thus, future studies with larger sample sizes and longer follow-up periods are warranted to validate these findings and evaluate their long-term clinical impact.

### Conclusions

This pilot randomized controlled trial demonstrated that a mobile app–based self-exercise program is feasible, acceptable, and safe for older patients with knee osteoarthritis. Over the 6-week intervention, participants in the mobile app group showed high adherence, strong satisfaction, and favorable trends in pain reduction and physical function compared with those using a paper-based program. By combining the proven benefits of exercise with the accessibility of digital technology, this approach shows promise for enhancing engagement and overcoming common barriers to exercise adherence. Future large-scale randomized controlled trials are warranted to confirm these preliminary findings and to evaluate the long-term effectiveness and clinical integration of mobile health interventions for knee osteoarthritis management.

## Supplementary material

10.2196/71073 Multimedia Appendix 1 Mobile app–based self-exercise program for patients with knee osteoarthritis.

10.2196/71073 Checklist 1 CONSORT-EHEALTH checklist.

## Abbreviations

30-CST: 30-second Chair Stand Test
CONSORT-EHEALTH: Consolidated Standards of Reporting Trials of Electronic and Mobile Health Applications and Online Telehealth
K-GDS: Korean version of the Geriatric Depression Scale
K-L: Kellgren-Lawrence
KOOS-PS: Knee Injury and Osteoarthritis Outcome Score–Physical Function Short form
NRS: Numeric Rating Scale
RCT: randomized controlled trial
SF-36: 36-Item Short-Form Health Survey
TUG: Timed Up-and-Go
WOMAC: Western Ontario and McMaster Universities Osteoarthritis Index
