# Supplementary material for: Feasibility and Preliminary Effectiveness of a Mobile App–Based Personalized Exercise Program in Older Patients With Chronic Knee Osteoarthritis: Pilot Randomized Controlled Trial
Source: JMIR Mhealth Uhealth. 2025 Dec 16;13:e71073. doi: 10.2196/71073 (PMC12707693; doi:10.2196/71073)
Supplement: Multimedia Appendix 1 [file mhealth-v13-e71073-s001.docx]

**Multimedia Appendix 1.** Mobile application–based self-exercise program for patients with knee osteoarthritis

|  | **Two stretching exercises** | **Three strengthening or functional exercises** | **Cooldown exercise and activity** |
| --- | --- | --- | --- |
| **Mild intensity** |  |  |  |
| Sunday | Hip adductor, overall lower extremity, and lumbosacral | Quadriceps, gastrocnemius, and mini (1/4) squat | Neck stretching (posterior), shoulder stretching, and go out for a walk |
| Monday | Gastrocnemius, overall lower extremity, and lumbosacral | Quadriceps, gastrocnemius, and lunge | Neck stretching (lateral), overall upper extremity stretching, and standing with eyes closed |
| Tuesday | Quadriceps and hamstring | Quadriceps, mini-squat, and lunge | Neck stretching (posterior), neck stretching (lateral), and go out for a walk |
| Wednesday | Hip adductor, overall lower extremity, and lumbosacral | Gastrocnemius, mini-squat, and lunge | Shoulder stretching, overall upper extremity stretching, and standing with eyes closed |
| Thursday | Quadriceps and gastrocnemius | Quadriceps, lunge, and mini-squat | Neck stretching (posterior), shoulder stretching, and go out for a walk |
| Friday | Hip adductor and hamstring | Mini-squat, lunge, and gastrocnemius | Overall upper extremity stretching, shoulder stretching, and standing with eyes closed |
| Saturday | Gastrocnemius, overall lower extremity, and lumbosacral | Gastrocnemius, lunge, and quadriceps | Neck stretching (posterior), overall upper extremity stretching, and go out for a walk |
| **Moderate intensity** |  |  |  |
| Sunday | Overall lower extremity and lumbosacral, quadriceps | Quadriceps, mini (1/4) squat, and lunge | Neck stretching (posterior), shoulder stretching, and go out for a walk |
| Monday | Hip adductor, overall lower extremity, and lumbosacral | Gastrocnemius, half (1/2) squat, and mini-squat | Neck stretching (lateral), overall upper extremity stretching, and stair up and down |
| Tuesday | Quadriceps and gastrocnemius | Mini-squat, half squat, and lunge | Neck stretching (posterior), neck stretching (lateral), and standing with eyes closed |
| Wednesday | Hamstring and hip adductor | Quadriceps, half squat, and lunge | Shoulder stretching, overall upper extremity stretching, and go out for a walk |
| Thursday | Quadriceps and gastrocnemius | Lunge, half squat, and gastrocnemius | Neck stretching (posterior), shoulder stretching, and stair up and down |
| Friday | Gastrocnemius, overall lower extremity, and lumbosacral | Gastrocnemius, half squat, and quadriceps | Overall upper extremity stretching, shoulder stretching, and standing with eyes closed |
| Saturday | Overall lower extremity and lumbosacral and quadriceps | Half squat, lunge, and mini-squat | Neck stretching (posterior), overall upper extremity stretching, and stair up and down |
| **High intensity** |  |  |  |
| Sunday | Hamstring and hip adductor | Bridge exercise, modified plank and quadriceps | Neck stretching (posterior), shoulder stretching, and go out for a walk |
| Monday | Overall lower extremity and lumbosacral, and quadriceps | Modified plank, gastrocnemius, and quadriceps | Neck stretching (lateral), overall upper extremity stretching, and stair up and down |
| Tuesday | Gastrocnemius, overall lower extremity and lumbosacral | Half squat, lunge, and bridge exercise | Neck stretching (posterior), neck stretching (lateral), and go out for a walk |
| Wednesday | Gastrocnemius and quadriceps | Lunge, gastrocnemius, and quadriceps | Shoulder stretching, overall upper extremity stretching, and stair up and down |
| Thursday | Overall lower extremity and lumbosacral and hamstring | Lunge, bridge exercise, and modified plank | Neck stretching (posterior), shoulder stretching, and go out for a walk |
| Friday | Hip adductor and gastrocnemius | Gastrocnemius, quadriceps, and half squat | Overall upper extremity stretching, shoulder stretching, and stair up and down |
| Saturday | Hamstring and gastrocnemius | Modified plank, lunge, and bridge exercise | Neck stretching (posterior), overall upper extremity stretching, and go out for a walk |
