# Supplementary material for: Feasibility and Preliminary Effectiveness of a Mobile App–Based Personalized Exercise Program in Older Patients With Chronic Knee Osteoarthritis: Pilot Randomized Controlled Trial
Source: JMIR Mhealth Uhealth. 2025 Dec 16;13:e71073. doi: 10.2196/71073 (PMC12707693; doi:10.2196/71073)
Supplement: Checklist 1 [file mhealth-v13-e71073-s002.pdf]

CONSORT-EHEALTH (V 1.6.1) - Submission/Publication Form

The CONSORT-EHEALTH checklist is intended for authors of randomized trials evaluating web-based and Internet-based applications/interventions, including mobile interventions, electronic games (incl multiplayer games), social media, certain telehealth applications, and other interactive and/or networked electronic applications. Some of the items (e.g. all subitems under item 5 - description of the intervention) may also be applicable for other study designs.

The goal of the CONSORT EHEALTH checklist and guideline is to be
a) a guide for reporting for authors of RCTs,
b) to form a basis for appraisal of an ehealth trial (in terms of validity)

CONSORT-EHEALTH items/subitems are MANDATORY reporting items for studies published in the Journal of Medical Internet Research and other journals / scientific societies endorsing the checklist.

Items numbered 1., 2., 3., 4a., 4b etc are original CONSORT or CONSORT-NPT (non-pharmacologic treatment) items.
Items with Roman numerals (i., ii, iii, iv etc.) are CONSORT-EHEALTH extensions/clarifications.

As the CONSORT-EHEALTH checklist is still considered in a formative stage, we would ask that you also RATE ON A SCALE OF 1-5 how important/useful you feel each item is FOR THE PURPOSE OF THE CHECKLIST and reporting guideline (optional).

Mandatory reporting items are marked with a red *.
In the textboxes, either copy & paste the relevant sections from your manuscript into this form - please include any quotes from your manuscript in QUOTATION MARKS,
or answer directly by providing additional information not in the manuscript, or elaborating on why the item was not relevant for this study.

YOUR ANSWERS WILL BE PUBLISHED AS A SUPPLEMENTARY FILE TO YOUR PUBLICATION IN JMIR AND ARE CONSIDERED PART OF YOUR PUBLICATION (IF ACCEPTED).
Please fill in these questions diligently. Information will not be copyedited, so please use proper spelling and grammar, use correct capitalization, and avoid abbreviations.

DO NOT FORGET TO SAVE AS PDF _AND_ CLICK THE SUBMIT BUTTON SO YOUR ANSWERS ARE IN OUR DATABASE !!!

Citation Suggestion (if you append the pdf as Appendix we suggest to cite this paper in the caption):
Eysenbach G, CONSORT-EHEALTH Group
CONSORT-EHEALTH: Improving and Standardizing Evaluation Reports of Web-based and Mobile Health Interventions
J Med Internet Res 2011;13(4):e126

URL: http://www.jmir.org/2011/4/e126/
doi: 10.2196/jmir.1923
PMID: 22209829

limyj9311@gmail.com 계정 전환

비공개

초안 저장됨

* 표시는 필수 질문임

Your name *

First Last

Yu Jin Im

Primary Affiliation (short), City, Country *

University of Toronto, Toronto, Canada

Department of Physical and Rehabilitation Me[illegible]

Your e-mail address *

abc@gmail.com

yujin93.im@samsung.com

Title of your manuscript *

Provide the (draft) title of your manuscript.

Effectiveness of a Mobile Application–Based Personalized Exercise Program in Elderly Patients with Chronic Knee Osteoarthritis: A Pilot Randomized Controlled Trial

Name of your App/Software/Intervention *

If there is a short and a long/alternate name, write the short name first and add the long name in brackets.

Mobile Application–Based Personalized Exerci

Evaluated Version (if any)

e.g. "V1", "Release 2017-03-01", "Version 2.0.27913"

V.1.6.1

Language(s) *

What language is the intervention/app in? If multiple languages are available, separate by comma (e.g. "English, French")

Korean

URL of your Intervention Website or App

e.g. a direct link to the mobile app on app in appstore (itunes, Google Play), or URL of the website. If the intervention is a DVD or hardware, you can also link to an Amazon page.

내 답변

URL of an image/screenshot (optional)

내 답변

Accessibility *

Can an enduser access the intervention presently?

- ○ access is free and open
- ○ access only for special usergroups, not open
- ○ access is open to everyone, but requires payment/subscription/in-app purchases
- ◉ app/intervention no longer accessible
- ○ 기타:

Primary Medical Indication/Disease/Condition *

e.g. "Stress", "Diabetes", or define the target group in brackets after the condition, e.g. "Autism (Parents of children with)", "Alzheimers (Informal Caregivers of)"

Chronic musculoskeletal disease (e.g. osteoar

Primary Outcomes measured in trial *

comma-separated list of primary outcomes reported in the trial

Western Ontario and McMaster Universities Os

Secondary/other outcomes

Are there any other outcomes the intervention is expected to affect?

Numeric Rating Scale (11-point scale) pain, Knee injury and Osteoarthritis Outcome Score–Physical Function Short form, Medical Outcomes Study 36-Item Short-Form Health Survey, Korean version of the Geriatric Depression Scale, Timed Up-and-Go test, 30-second Chair Stand Test, handgrip strength test, and knee extensor strength test

Recommended "Dose" *

What do the instructions for users say on how often the app should be used?

- (●) Approximately Daily
- ( ) Approximately Weekly
- ( ) Approximately Monthly
- ( ) Approximately Yearly
- ( ) "as needed"
- ( ) 기타:

Approx. Percentage of Users (starters) still using the app as recommended after 3 months *

- (●) unknown / not evaluated
- ( ) 0-10%
- ( ) 11-20%
- ( ) 21-30%
- ( ) 31-40%
- ( ) 41-50%
- ( ) 51-60%
- ( ) 61-70%
- ( ) 71%-80%
- ( ) 81-90%
- ( ) 91-100%
- ( ) 기타:

Overall, was the app/intervention effective? *

- (x) yes: all primary outcomes were significantly better in intervention group vs control
- ( ) partly: SOME primary outcomes were significantly better in intervention group vs control
- ( ) no statistically significant difference between control and intervention
- ( ) potentially harmful: control was significantly better than intervention in one or more outcomes
- ( ) inconclusive: more research is needed
- ( ) 기타:

Article Preparation Status/Stage *

At which stage in your article preparation are you currently (at the time you fill in this form)

- ( ) not submitted yet - in early draft status
- (x) not submitted yet - in late draft status, just before submission
- ( ) submitted to a journal but not reviewed yet
- ( ) submitted to a journal and after receiving initial reviewer comments
- ( ) submitted to a journal and accepted, but not published yet
- ( ) published
- ( ) 기타:

Journal *

If you already know where you will submit this paper (or if it is already submitted), please provide the journal name (if it is not JMIR, provide the journal name under "other")

- ○ not submitted yet / unclear where I will submit this
- ○ Journal of Medical Internet Research (JMIR)
- ◉ JMIR mHealth and UHealth
- ○ JMIR Serious Games
- ○ JMIR Mental Health
- ○ JMIR Public Health
- ○ JMIR Formative Research
- ○ Other JMIR sister journal
- ○ 기타:

Is this a full powered effectiveness trial or a pilot/feasibility trial? *

- ◉ Pilot/feasibility
- ○ Fully powered

Manuscript tracking number *

If this is a JMIR submission, please provide the manuscript tracking number under "other" (The ms tracking number can be found in the submission acknowledgement email, or when you login as author in JMIR. If the paper is already published in JMIR, then the ms tracking number is the four-digit number at the end of the DOI, to be found at the bottom of each published article in JMIR)

- ◉ no ms number (yet) / not (yet) submitted to / published in JMIR
- ○ 기타:

TITLE AND ABSTRACT

1a) TITLE: Identification as a randomized trial in the title

1a) Does your paper address CONSORT item 1a? *

I.e does the title contain the phrase "Randomized Controlled Trial"? (if not, explain the reason under "other")

- (●) yes
- ( ) 기타:

1a-i) Identify the mode of delivery in the title

Identify the mode of delivery. Preferably use “web-based” and/or “mobile” and/or “electronic game” in the title. Avoid ambiguous terms like “online”, “virtual”, “interactive”. Use “Internet-based” only if Intervention includes non-web-based Internet components (e.g. email), use “computer-based” or “electronic” only if offline products are used. Use “virtual” only in the context of “virtual reality” (3-D worlds). Use “online” only in the context of “online support groups”. Complement or substitute product names with broader terms for the class of products (such as “mobile” or “smart phone” instead of “iphone”), especially if the application runs on different platforms.

	1	2	3	4	5	
subitem not at all important	( )	( )	( )	( )	(●)	essential

선택해제

Does your paper address subitem 1a-i? *

Copy and paste relevant sections from manuscript title (include quotes in quotation marks "like this" to indicate direct quotes from your manuscript), or elaborate on this item by providing additional information not in the ms, or briefly explain why the item is not applicable/relevant for your study

"Mobile Application–Based Personalized Exercise Program"

1a-ii) Non-web-based components or important co-interventions in title

Mention non-web-based components or important co-interventions in title, if any (e.g., “with telephone support”).

	1	2	3	4	5	
subitem not at all important	○	○	◉	○	○	essential

선택해제

Does your paper address subitem 1a-ii?

Copy and paste relevant sections from manuscript title (include quotes in quotation marks "like this" to indicate direct quotes from your manuscript), or elaborate on this item by providing additional information not in the ms, or briefly explain why the item is not applicable/relevant for your study

This subitem is not applicable to our study, as we used only a mobile application-based intervention without any co-interventions.

1a-iii) Primary condition or target group in the title

Mention primary condition or target group in the title, if any (e.g., “for children with Type I Diabetes”) Example: A Web-based and Mobile Intervention with Telephone Support for Children with Type I Diabetes: Randomized Controlled Trial

	1	2	3	4	5	
subitem not at all important	○	○	○	○	◉	essential

선택해제

Does your paper address subitem 1a-iii? *

Copy and paste relevant sections from manuscript title (include quotes in quotation marks "like this" to indicate direct quotes from your manuscript), or elaborate on this item by providing additional information not in the ms, or briefly explain why the item is not applicable/relevant for your study

"in Elderly Patients with Chronic Knee Osteoarthritis"

1b) ABSTRACT: Structured summary of trial design, methods, results, and conclusions

NPT extension: Description of experimental treatment, comparator, care providers, centers, and blinding status.

1b-i) Key features/functionalities/components of the intervention and comparator in the METHODS section of the ABSTRACT

Mention key features/functionalities/components of the intervention and comparator in the abstract. If possible, also mention theories and principles used for designing the site. Keep in mind the needs of systematic reviewers and indexers by including important synonyms. (Note: Only report in the abstract what the main paper is reporting. If this information is missing from the main body of text, consider adding it)

	1	2	3	4	5	
subitem not at all important	○	○	○	○	◉	essential

선택해제

Does your paper address subitem 1b-i? *

Copy and paste relevant sections from the manuscript abstract (include quotes in quotation marks "like this" to indicate direct quotes from your manuscript), or elaborate on this item by providing additional information not in the ms, or briefly explain why the item is not applicable/relevant for your study

"randomized at a 2:1 ratio to either the intervention (n = 19; mobile application–based program) or control (n = 10; paper-based program)"

1b-ii) Level of human involvement in the METHODS section of the ABSTRACT

Clarify the level of human involvement in the abstract, e.g., use phrases like “fully automated” vs. “therapist/nurse/care provider/physician-assisted” (mention number and expertise of providers involved, if any). (Note: Only report in the abstract what the main paper is reporting. If this information is missing from the main body of text, consider adding it)

	1	2	3	4	5	
subitem not at all important	○	○	○	○	◉	essential

선택해제

Does your paper address subitem 1b-ii?

Copy and paste relevant sections from the manuscript abstract (include quotes in quotation marks "like this" to indicate direct quotes from your manuscript), or elaborate on this item by providing additional information not in the ms, or briefly explain why the item is not applicable/relevant for your study

"In this pilot, single-blinded, randomized controlled trial, 29 participants aged ≥ 60 years with chronic knee pain and radiographic evidence of OA were randomized at a 2:1 ratio to either the intervention (n = 19; mobile application–based program) or control (n = 10; paper-based program) group. The mobile application delivered a personalized exercise program, which was tailored by physiotherapists based on remote monitoring of patient-reported symptoms.
"

1b-iii) Open vs. closed, web-based (self-assessment) vs. face-to-face assessments in the METHODS section of the ABSTRACT

Mention how participants were recruited (online vs. offline), e.g., from an open access website or from a clinic or a closed online user group (closed usergroup trial), and clarify if this was a purely web-based trial, or there were face-to-face components (as part of the intervention or for assessment). Clearly say if outcomes were self-assessed through questionnaires (as common in web-based trials). Note: In traditional offline trials, an open trial (open-label trial) is a type of clinical trial in which both the researchers and participants know which treatment is being administered. To avoid confusion, use “blinded” or “unblinded” to indicated the level of blinding instead of “open”, as “open” in web-based trials usually refers to “open access” (i.e. participants can self-enrol). (Note: Only report in the abstract what the main paper is reporting. If this information is missing from the main body of text, consider adding it)

	1	2	3	4	5	
subitem not at all important	○	○	◉	○	○	essential

선택해제

Does your paper address subitem 1b-iii?

Copy and paste relevant sections from the manuscript abstract (include quotes in quotation marks "like this" to indicate direct quotes from your manuscript), or elaborate on this item by providing additional information not in the ms, or briefly explain why the item is not applicable/relevant for your study

"Methods: In this pilot, single-blinded, randomized controlled trial, 29 participants aged ≥ 60 years with chronic knee pain and radiographic evidence of OA were randomized at a 2:1 ratio to either the intervention (n = 19; mobile application–based program) or control (n = 10; paper-based program) group."

1b-iv) RESULTS section in abstract must contain use data

Report number of participants enrolled/assessed in each group, the use/uptake of the intervention (e.g., attrition/adherence metrics, use over time, number of logins etc.), in addition to primary/secondary outcomes. (Note: Only report in the abstract what the main paper is reporting. If this information is missing from the main body of text, consider adding it)

	1	2	3	4	5	
subitem not at all important	○	○	○	○	◉	essential

선택해제

Does your paper address subitem 1b-iv?

Copy and paste relevant sections from the manuscript abstract (include quotes in quotation marks "like this" to indicate direct quotes from your manuscript), or elaborate on this item by providing additional information not in the ms, or briefly explain why the item is not applicable/relevant for your study

"29 participants aged ≥ 60 years with chronic knee pain and radiographic evidence of OA were randomized"

1b-v) CONCLUSIONS/DISCUSSION in abstract for negative trials

Conclusions/Discussions in abstract for negative trials: Discuss the primary outcome - if the trial is negative (primary outcome not changed), and the intervention was not used, discuss whether negative results are attributable to lack of uptake and discuss reasons. (Note: Only report in the abstract what the main paper is reporting. If this information is missing from the main body of text, consider adding it)

	1	2	3	4	5	
subitem not at all important	○	○	○	○	◉	essential

선택해제

Does your paper address subitem 1b-v?

Copy and paste relevant sections from the manuscript abstract (include quotes in quotation marks "like this" to indicate direct quotes from your manuscript), or elaborate on this item by providing additional information not in the ms, or briefly explain why the item is not applicable/relevant for your study

"The intervention group exhibited significant reductions in the WOMAC total score (median change: −11.00; 95% confidence interval [CI]: −23.00 to −2.50; P=.01) and NRS pain score (mean change: −2.12; 95% CI: −3.13 to −1.11; P<.001)."

INTRODUCTION

2a) In INTRODUCTION: Scientific background and explanation of rationale

2a-i) Problem and the type of system/solution

Describe the problem and the type of system/solution that is object of the study: intended as stand-alone intervention vs. incorporated in broader health care program? Intended for a particular patient population? Goals of the intervention, e.g., being more cost-effective to other interventions, replace or complement other solutions? (Note: Details about the intervention are provided in “Methods” under 5)

	1	2	3	4	5	
subitem not at all important	○	○	○	○	◉	essential

선택해제

Does your paper address subitem 2a-i? *

Copy and paste relevant sections from the manuscript (include quotes in quotation marks "like this" to indicate direct quotes from your manuscript), or elaborate on this item by providing additional information not in the ms, or briefly explain why the item is not applicable/relevant for your study

"Exercise interventions effectively reduce pain and improve function in patients with knee OA [6-9]. Considering the chronic nature of knee OA and its frequent association with comorbidities, such as cardiovascular disease and diabetes mellitus, nonpharmacologic strategies, including patient education and exercise, are crucial for long-term self-management of knee OA [1]. Furthermore, advancements in digital technology and widespread adoption of mobile devices have expanded the delivery modes of exercise programs. Among these, mobile application–based self-exercise programs have emerged as cost-effective solutions for overcoming barriers such as limited access to healthcare facilities [10,11]. Furthermore, mobile applications, offering video-guided exercises and remote monitoring systems can improve exercise adherence and the effectiveness of self-directed exercise."

2a-ii) Scientific background, rationale: What is known about the (type of) system

Scientific background, rationale: What is known about the (type of) system that is the object of the study (be sure to discuss the use of similar systems for other conditions/diagnoses, if appropiate), motivation for the study, i.e. what are the reasons for and what is the context for this specific study, from which stakeholder viewpoint is the study performed, potential impact of findings [2]. Briefly justify the choice of the comparator.

	1	2	3	4	5	
subitem not at all important	○	○	○	○	◉	essential

선택해제

Does your paper address subitem 2a-ii? *

Copy and paste relevant sections from the manuscript (include quotes in quotation marks "like this" to indicate direct quotes from your manuscript), or elaborate on this item by providing additional information not in the ms, or briefly explain why the item is not applicable/relevant for your study

"Although several randomized controlled trials on web- or mobile-based exercise programs have been conducted for managing knee OA, the number of studies on mobile applications that integrate regular patient-reported symptom assessment and dynamically adjust exercise programs based on remote monitoring are limited [12-21]."

2b) In INTRODUCTION: Specific objectives or hypotheses

Does your paper address CONSORT subitem 2b? *

Copy and paste relevant sections from the manuscript (include quotes in quotation marks "like this" to indicate direct quotes from your manuscript), or elaborate on this item by providing additional information not in the ms, or briefly explain why the item is not applicable/relevant for your study

"This study evaluated the feasibility and efficacy of a 6-week mobile application–based self-exercise program with a multimonitoring system in improving pain, physical function, and muscle strength in patients with knee OA and compared them with those of a conventional paper-based self-exercise program. We hypothesized that our mobile application–based self-exercise program will demonstrate superior outcomes compared with the paper-based self-exercise program."

METHODS

3a) Description of trial design (such as parallel, factorial) including allocation ratio

Does your paper address CONSORT subitem 3a? *

Copy and paste relevant sections from the manuscript (include quotes in quotation marks "like this" to indicate direct quotes from your manuscript), or elaborate on this item by providing additional information not in the ms, or briefly explain why the item is not applicable/relevant for your study

"Trial Design
This prospective, parallel, two-armed, single-blinded, single-center, randomized controlled trial was prospectively registered at ClinicalTrials.gov (NCT05197010) and approved by the Institutional Review Board of Samsung Medical Center (2021-04-004)." "Randomization
Eligible participants were randomized to the intervention or control group in a 2:1 ratio using block randomization with a block size of 6. "

3b) Important changes to methods after trial commencement (such as eligibility criteria), with reasons

Does your paper address CONSORT subitem 3b? *

Copy and paste relevant sections from the manuscript (include quotes in quotation marks "like this" to indicate direct quotes from your manuscript), or elaborate on this item by providing additional information not in the ms, or briefly explain why the item is not applicable/relevant for your study

This subitem is not applicable to our study because there was no change.

3b-i) Bug fixes, Downtimes, Content Changes

Bug fixes, Downtimes, Content Changes: ehealth systems are often dynamic systems. A description of changes to methods therefore also includes important changes made on the intervention or comparator during the trial (e.g., major bug fixes or changes in the functionality or content) (5-iii) and other “unexpected events” that may have influenced study design such as staff changes, system failures/downtimes, etc. [2].

	1	2	3	4	5	
subitem not at all important	◉	○	○	○	○	essential

선택해제

Does your paper address subitem 3b-i?

Copy and paste relevant sections from the manuscript (include quotes in quotation marks "like this" to indicate direct quotes from your manuscript), or elaborate on this item by providing additional information not in the ms, or briefly explain why the item is not applicable/relevant for your study

내 답변

4a) Eligibility criteria for participants

Does your paper address CONSORT subitem 4a? *

Copy and paste relevant sections from the manuscript (include quotes in quotation marks "like this" to indicate direct quotes from your manuscript), or elaborate on this item by providing additional information not in the ms, or briefly explain why the item is not applicable/relevant for your study

"Inclusion criteria were: (1) age ≥ 60 years; (2) knee pain persisting for ≥ 3 months; (3) overall knee pain severity ≥ 4 on 11-point numeric rating scale (NRS); (4) score ≥ 23 points on the Korean version of the Montreal Cognitive Assessment (K-MoCA) [24], indicating ability to comprehend exercise program; and (5) radiographic evidence of osteoarthritis with Kellgren–Lawrence (K–L) grade ≥ 2 [25]. "

4a-i) Computer / Internet literacy

Computer / Internet literacy is often an implicit “de facto” eligibility criterion - this should be explicitly clarified.

	1	2	3	4	5	
subitem not at all important	○	○	○	○	◉	essential

선택해제

Does your paper address subitem 4a-i?

Copy and paste relevant sections from the manuscript (include quotes in quotation marks "like this" to indicate direct quotes from your manuscript), or elaborate on this item by providing additional information not in the ms, or briefly explain why the item is not applicable/relevant for your study

"(4) score ≥ 23 points on the Korean version of the Montreal Cognitive Assessment (K-MoCA) [24], indicating ability to comprehend exercise program; "

4a-ii) Open vs. closed, web-based vs. face-to-face assessments:

Open vs. closed, web-based vs. face-to-face assessments: Mention how participants were recruited (online vs. offline), e.g., from an open access website or from a clinic, and clarify if this was a purely web-based trial, or there were face-to-face components (as part of the intervention or for assessment), i.e., to what degree got the study team to know the participant. In online-only trials, clarify if participants were quasi-anonymous and whether having multiple identities was possible or whether technical or logistical measures (e.g., cookies, email confirmation, phone calls) were used to detect/prevent these.

	1	2	3	4	5	
subitem not at all important	○	○	○	○	◉	essential

선택해제

Does your paper address subitem 4a-ii? *

Copy and paste relevant sections from the manuscript (include quotes in quotation marks "like this" to indicate direct quotes from your manuscript), or elaborate on this item by providing additional information not in the ms, or briefly explain why the item is not applicable/relevant for your study

"The primary outcome was the total score of the Western Ontario and McMaster Universities Osteoarthritis Index (WOMAC) [26,27]. The secondary outcomes included knee pain assessed using an NRS (11-point scale), KOOS–PS [22,23], Medical Outcomes Study 36-Item Short-Form Health Survey (SF-36) [28,29], Korean version of the Geriatric Depression Scale (K-GDS) [30,31], Timed Up-and-Go (TUG) test [32], 30-second Chair Stand Test (30-CST) [33], handgrip strength test, and knee extensor strength test. Furthermore, quadriceps femoris muscle thickness was measured via ultrasonographic evaluation. The primary and secondary outcomes were evaluated at baseline and after 6 weeks. Physical function tests were assessed by an experienced physical therapist (S.A.C.), and ultrasound examinations were performed by an experienced physiatrist (J.G.D.). Adverse events were monitored throughout the study period."

4a-iii) Information giving during recruitment

Information given during recruitment. Specify how participants were briefed for recruitment and in the informed consent procedures (e.g., publish the informed consent documentation as appendix, see also item X26), as this information may have an effect on user self-selection, user expectation and may also bias results.

	1	2	3	4	5	
subitem not at all important	○	○	○	○	◉	essential

선택해제

Does your paper address subitem 4a-iii?

Copy and paste relevant sections from the manuscript (include quotes in quotation marks "like this" to indicate direct quotes from your manuscript), or elaborate on this item by providing additional information not in the ms, or briefly explain why the item is not applicable/relevant for your study

"Participants
The study participants were recruited from the musculoskeletal clinic of a tertiary academic medical center between October 2021 and December 2021. Informed consent was obtained from all participants before screening. "

4b) Settings and locations where the data were collected

Does your paper address CONSORT subitem 4b? *

Copy and paste relevant sections from the manuscript (include quotes in quotation marks "like this" to indicate direct quotes from your manuscript), or elaborate on this item by providing additional information not in the ms, or briefly explain why the item is not applicable/relevant for your study

"Participants
The study participants were recruited from the musculoskeletal clinic of a tertiary academic medical center between October 2021 and December 2021. Informed consent was obtained from all participants before screening. "

4b-i) Report if outcomes were (self-)assessed through online questionnaires

Clearly report if outcomes were (self-)assessed through online questionnaires (as common in web-based trials) or otherwise.

	1	2	3	4	5	
subitem not at all important	○	○	○	○	◉	essential

선택해제

Does your paper address subitem 4b-i? *

Copy and paste relevant sections from the manuscript (include quotes in quotation marks "like this" to indicate direct quotes from your manuscript), or elaborate on this item by providing additional information not in the ms, or briefly explain why the item is not applicable/relevant for your study

"To monitor the progress and adjust the intensity and content of the exercise program, the application incorporated a patient-reporting system. Before each daily exercise session, the participants quantified their pain intensity using a five-point Likert scale. After completion, they again rated their perceived exertion using a five-point Likert scale. On a weekly basis, the participants completed the KOOS−PS within the application. Accordingly, the application provided immediate visual feedback to the participants, and participants could check their weekly statistics for exercise achievement and pain levels within the application (Figure 1). "

4b-ii) Report how institutional affiliations are displayed

Report how institutional affiliations are displayed to potential participants [on ehealth media], as affiliations with prestigious hospitals or universities may affect volunteer rates, use, and reactions with regards to an intervention.(Not a required item – describe only if this may bias results)

	1	2	3	4	5	
subitem not at all important	◉	○	○	○	○	essential

선택해제

Does your paper address subitem 4b-ii?

Copy and paste relevant sections from the manuscript (include quotes in quotation marks "like this" to indicate direct quotes from your manuscript), or elaborate on this item by providing additional information not in the ms, or briefly explain why the item is not applicable/relevant for your study

Not relevant to our study; Institutional affiliations were not displayed to participants.

5) The interventions for each group with sufficient details to allow replication, including how and when they were actually administered

5-i) Mention names, credential, affiliations of the developers, sponsors, and owners

Mention names, credential, affiliations of the developers, sponsors, and owners [6] (if authors/evaluators are owners or developer of the software, this needs to be declared in a "Conflict of interest" section or mentioned elsewhere in the manuscript).

	1	2	3	4	5	
subitem not at all important	○	○	○	○	◉	essential

선택해제

Does your paper address subitem 5-i?

Copy and paste relevant sections from the manuscript (include quotes in quotation marks "like this" to indicate direct quotes from your manuscript), or elaborate on this item by providing additional information not in the ms, or briefly explain why the item is not applicable/relevant for your study

"Acknowledgements
This work was supported by the Technology Innovation Program (20003658), funded by the Ministry of Trade, Industry & Energy (Republic of Korea). The funders had no role in the study design, collection, analysis, and interpretation of data."

5-ii) Describe the history/development process

Describe the history/development process of the application and previous formative evaluations (e.g., focus groups, usability testing), as these will have an impact on adoption/use rates and help with interpreting results.

	1	2	3	4	5	
subitem not at all important	○	○	○	◉	○	essential

선택해제

Does your paper address subitem 5-ii?

Copy and paste relevant sections from the manuscript (include quotes in quotation marks "like this" to indicate direct quotes from your manuscript), or elaborate on this item by providing additional information not in the ms, or briefly explain why the item is not applicable/relevant for your study

"The intervention group participants underwent a 6-week self-exercise program delivered via a mobile application based self-exercise program (Figure 1). During their initial visit, participants were instructed to install the application on their mobile devices as well as received comprehensive training on its use. The application guided users through a daily 30-min self-exercise protocol, comprising stretching, strengthening and functional, and cooldown exercises (Multimedia Appendix 1). The exercise program was tailored by physiotherapists based on the patient's pain scale, functional status, and overall condition, ensuring a personalized approach to rehabilitation. To monitor the progress and adjust the intensity and content of the exercise program, the application incorporated a patient-reporting system. Before each daily exercise session, the participants quantified their pain intensity using a five-point Likert scale. After completion, they again rated their perceived exertion using a five-point Likert scale. On a weekly basis, the participants completed the KOOS–PS within the application. Accordingly, the application provided immediate visual feedback to the participants, and participants could check their weekly statistics for exercise achievement and pain levels within the application (Figure 1). Further, these data were transmitted to the supervising physiotherapist and physiatrist for remote monitoring and exercise program modifications. The exercise program was adjusted weekly to one of the three intensity levels (i.e., high, moderate, and mild) based on patient-reported outcomes (Figure 2). Owing to this approach, the application delivered a personalized self-exercise program throughout the 6-week intervention period."

5-iii) Revisions and updating

Revisions and updating. Clearly mention the date and/or version number of the application/intervention (and comparator, if applicable) evaluated, or describe whether the intervention underwent major changes during the evaluation process, or whether the development and/or content was “frozen” during the trial. Describe dynamic components such as news feeds or changing content which may have an impact on the replicability of the intervention (for unexpected events see item 3b).

	1	2	3	4	5	
subitem not at all important	(●)	○	○	○	○	essential

선택해제

Does your paper address subitem 5-iii?

Copy and paste relevant sections from the manuscript (include quotes in quotation marks "like this" to indicate direct quotes from your manuscript), or elaborate on this item by providing additional information not in the ms, or briefly explain why the item is not applicable/relevant for your study

내 답변

5-iv) Quality assurance methods

Provide information on quality assurance methods to ensure accuracy and quality of information provided [1], if applicable.

	1	2	3	4	5	
subitem not at all important	○	○	○	○	(●)	essential

선택해제

Does your paper address subitem 5-iv?

Copy and paste relevant sections from the manuscript (include quotes in quotation marks "like this" to indicate direct quotes from your manuscript), or elaborate on this item by providing additional information not in the ms, or briefly explain why the item is not applicable/relevant for your study

"All analyses were performed using R (version 4.2.1; R Foundation for Statistical Computing, Vienna, Austria) along with a modified intention-to-treat approach. The Shapiro–Wilk test was used to assess data normality. Between-group comparisons of baseline characteristics and outcome measures at baseline and at the 6-week follow-up were performed using Fisher's exact test for categorical variables. For continuous variables, either the independent t-tests or Wilcoxon rank-sum test was used, based on the normality of the data.

The primary efficacy analysis evaluated the between-group differences in changes from baseline to 6-week follow-up. Between-group differences in changes were analyzed using independent t-tests with mean differences and 95% confidence interval (CI) when normality assumptions were met. Otherwise, the Wilcoxon rank-sum test was performed, and the median differences with 95% CI were estimated using the Hodges–Lehmann method. For within-group comparisons, paired t-test or Wilcoxon signed-rank test was performed based on the normality of the data."

5-v) Ensure replicability by publishing the source code, and/or providing screenshots/screen-capture video, and/or providing flowcharts of the algorithms used

Ensure replicability by publishing the source code, and/or providing screenshots/screen-capture video, and/or providing flowcharts of the algorithms used. Replicability (i.e., other researchers should in principle be able to replicate the study) is a hallmark of scientific reporting.

	1	2	3	4	5	
subitem not at all important	○	○	○	◉	○	essential

선택해제

Does your paper address subitem 5-v?

Copy and paste relevant sections from the manuscript (include quotes in quotation marks "like this" to indicate direct quotes from your manuscript), or elaborate on this item by providing additional information not in the ms, or briefly explain why the item is not applicable/relevant for your study

"Figure 1. Features of the mobile application–based interactive exercise program
The application is designed to facilitate self-managed knee rehabilitation via the following features: (A) instructional videos guiding users for performing prescribed exercises accurately within the application interface; (B) daily self-assessment of pain levels using a visual pain scale to monitor symptoms before exercise; (C) weekly tracking of self-reported outcomes, including functional difficulties due to knee pain, measured using the Knee Injury and Osteoarthritis Outcome Score–Physical Function Short Form; and (D) visualized statistics summarizing user progress, including weekly exercise achievement rates and pain indices, offering insights into rehabilitation progress over time.

Figure 2. Exercise adjustment interface in mobile application for clinicians
The application provides a platform for physical therapists and physiatrists to remotely monitor weekly patient-reported outcomes and assess the effectiveness of the current exercise program. Clinicians can review the patient's ongoing exercise regimen and adjust the exercise intensity (mild, moderate, or high) accordingly. "

5-vi) Digital preservation

Digital preservation: Provide the URL of the application, but as the intervention is likely to change or disappear over the course of the years; also make sure the intervention is archived (Internet Archive, webcitation.org, and/or publishing the source code or screenshots/videos alongside the article). As pages behind login screens cannot be archived, consider creating demo pages which are accessible without login.

	1	2	3	4	5	
subitem not at all important	○	○	◉	○	○	essential

선택해제

Does your paper address subitem 5-vi?

Copy and paste relevant sections from the manuscript (include quotes in quotation marks "like this" to indicate direct quotes from your manuscript), or elaborate on this item by providing additional information not in the ms, or briefly explain why the item is not applicable/relevant for your study

In our study, the URL of the application was not included.

5-vii) Access

Access: Describe how participants accessed the application, in what setting/context, if they had to pay (or were paid) or not, whether they had to be a member of specific group. If known, describe how participants obtained “access to the platform and Internet” [1]. To ensure access for editors/reviewers/readers, consider to provide a “backdoor” login account or demo mode for reviewers/readers to explore the application (also important for archiving purposes, see vi).

	1	2	3	4	5	
subitem not at all important	(●)	( )	( )	( )	( )	essential

선택해제

Does your paper address subitem 5-vii? *

Copy and paste relevant sections from the manuscript (include quotes in quotation marks "like this" to indicate direct quotes from your manuscript), or elaborate on this item by providing additional information not in the ms, or briefly explain why the item is not applicable/relevant for your study

"The intervention group participants underwent a 6-week self-exercise program delivered via a mobile application based self-exercise program (Figure 1). During their initial visit, participants were instructed to install the application on their mobile devices as well as received comprehensive training on its use. "

5-viii) Mode of delivery, features/functionalities/components of the intervention and comparator, and the theoretical framework

Describe mode of delivery, features/functionalities/components of the intervention and comparator, and the theoretical framework [6] used to design them (instructional strategy [1], behaviour change techniques, persuasive features, etc., see e.g., [7, 8] for terminology). This includes an in-depth description of the content (including where it is coming from and who developed it) [1]," whether [and how] it is tailored to individual circumstances and allows users to track their progress and receive feedback" [6]. This also includes a description of communication delivery channels and – if computer-mediated communication is a component – whether communication was synchronous or asynchronous [6]. It also includes information on presentation strategies [1], including page design principles, average amount of text on pages, presence of hyperlinks to other resources, etc. [1].

	1	2	3	4	5	
subitem not at all important	○	○	○	○	◉	essential

선택해제

Does your paper address subitem 5-viii? *

Copy and paste relevant sections from the manuscript (include quotes in quotation marks "like this" to indicate direct quotes from your manuscript), or elaborate on this item by providing additional information not in the ms, or briefly explain why the item is not applicable/relevant for your study

"Intervention Group
The intervention group participants underwent a 6-week self-exercise program delivered via a mobile application based self-exercise program (Figure 1). During their initial visit, participants were instructed to install the application on their mobile devices as well as received comprehensive training on its use. The application guided users through a daily 30-min self-exercise protocol, comprising stretching, strengthening and functional, and cooldown exercises (Multimedia Appendix 1). The exercise program was tailored by physiotherapists based on the patient's pain scale, functional status, and overall condition, ensuring a personalized approach to rehabilitation. To monitor the progress and adjust the intensity and content of the exercise program, the application incorporated a patient-reporting system. Before each daily exercise session, the participants quantified their pain intensity using a five-point Likert scale. After completion, they again rated their perceived exertion using a five-point Likert scale. On a weekly basis, the participants completed the KOOS–PS within the application. Accordingly, the application provided immediate visual feedback to the participants, and participants could check their weekly statistics for exercise achievement and pain levels within the application (Figure 1). Further, these data were transmitted to the supervising physiotherapist and physiatrist for remote monitoring and exercise program modifications. The exercise program was adjusted weekly to one of the three intensity levels (i.e., high, moderate, and mild) based on patient-reported outcomes (Figure 2). Owing to this approach, the application delivered a personalized self-exercise program throughout the 6-week intervention period."

5-ix) Describe use parameters

Describe use parameters (e.g., intended “doses” and optimal timing for use). Clarify what instructions or recommendations were given to the user, e.g., regarding timing, frequency, heaviness of use, if any, or was the intervention used ad libitum.

	1	2	3	4	5	
subitem not at all important	○	○	○	○	◉	essential

선택해제

Does your paper address subitem 5-ix?

Copy and paste relevant sections from the manuscript (include quotes in quotation marks "like this" to indicate direct quotes from your manuscript), or elaborate on this item by providing additional information not in the ms, or briefly explain why the item is not applicable/relevant for your study

"Intervention Group
The intervention group participants underwent a 6-week self-exercise program delivered via a mobile application based self-exercise program (Figure 1). During their initial visit, participants were instructed to install the application on their mobile devices as well as received comprehensive training on its use. The application guided users through a daily 30-min self-exercise protocol, comprising stretching, strengthening and functional, and cooldown exercises (Multimedia Appendix 1). The exercise program was tailored by physiotherapists based on the patient's pain scale, functional status, and overall condition, ensuring a personalized approach to rehabilitation. To monitor the progress and adjust the intensity and content of the exercise program, the application incorporated a patient-reporting system. Before each daily exercise session, the participants quantified their pain intensity using a five-point Likert scale. After completion, they again rated their perceived exertion using a five-point Likert scale. On a weekly basis, the participants completed the KOOS–PS within the application. Accordingly, the application provided immediate visual feedback to the participants, and participants could check their weekly statistics for exercise achievement and pain levels within the application (Figure 1). Further, these data were transmitted to the supervising physiotherapist and physiatrist for remote monitoring and exercise program modifications. The exercise program was adjusted weekly to one of the three intensity levels (i.e., high, moderate, and mild) based on patient-reported outcomes (Figure 2). Owing to this approach, the application delivered a personalized self-exercise program throughout the 6-week intervention period."

5-x) Clarify the level of human involvement

Clarify the level of human involvement (care providers or health professionals, also technical assistance) in the e-intervention or as co-intervention (detail number and expertise of professionals involved, if any, as well as “type of assistance offered, the timing and frequency of the support, how it is initiated, and the medium by which the assistance is delivered”. It may be necessary to distinguish between the level of human involvement required for the trial, and the level of human involvement required for a routine application outside of a RCT setting (discuss under item 21 – generalizability).

	1	2	3	4	5	
subitem not at all important	○	○	○	○	◉	essential

선택해제

Does your paper address subitem 5-x?

Copy and paste relevant sections from the manuscript (include quotes in quotation marks "like this" to indicate direct quotes from your manuscript), or elaborate on this item by providing additional information not in the ms, or briefly explain why the item is not applicable/relevant for your study

"Intervention Group
The intervention group participants underwent a 6-week self-exercise program delivered via a mobile application based self-exercise program (Figure 1). During their initial visit, participants were instructed to install the application on their mobile devices as well as received comprehensive training on its use. The application guided users through a daily 30-min self-exercise protocol, comprising stretching, strengthening and functional, and cooldown exercises (Multimedia Appendix 1). The exercise program was tailored by physiotherapists based on the patient's pain scale, functional status, and overall condition, ensuring a personalized approach to rehabilitation. To monitor the progress and adjust the intensity and content of the exercise program, the application incorporated a patient-reporting system. Before each daily exercise session, the participants quantified their pain intensity using a five-point Likert scale. After completion, they again rated their perceived exertion using a five-point Likert scale. On a weekly basis, the participants completed the KOOS–PS within the application. Accordingly, the application provided immediate visual feedback to the participants, and participants could check their weekly statistics for exercise achievement and pain levels within the application (Figure 1). Further, these data were transmitted to the supervising physiotherapist and physiatrist for remote monitoring and exercise program modifications. The exercise program was adjusted weekly to one of the three intensity levels (i.e., high, moderate, and mild) based on patient-reported outcomes (Figure 2). Owing to this approach, the application delivered a personalized self-exercise program throughout the 6-week intervention period."

5-xi) Report any prompts/reminders used

Report any prompts/reminders used: Clarify if there were prompts (letters, emails, phone calls, SMS) to use the application, what triggered them, frequency etc. It may be necessary to distinguish between the level of prompts/reminders required for the trial, and the level of prompts/reminders for a routine application outside of a RCT setting (discuss under item 21 – generalizability).

	1	2	3	4	5	
subitem not at all important	○	○	○	○	◉	essential

선택해제

Does your paper address subitem 5-xi? *

Copy and paste relevant sections from the manuscript (include quotes in quotation marks "like this" to indicate direct quotes from your manuscript), or elaborate on this item by providing additional information not in the ms, or briefly explain why the item is not applicable/relevant for your study

"The success of the application in maintaining adherence can be attributed to three key features: first, automated pop-up alarms delivered via participants' mobile phones are active reminders to encourage consistent participation in the exercise program. Second, the visualized statistical tracking features and weekly patient-reported symptom monitoring system of the application enable participants to continuously assess their progress, providing tangible feedback on their exercise achievements. Third, the dynamic modification of exercise intensity based on individual progression would maintain appropriate challenge levels while accommodating participants' changing capabilities. "

5-xii) Describe any co-interventions (incl. training/support)

Describe any co-interventions (incl. training/support): Clearly state any interventions that are provided in addition to the targeted eHealth intervention, as ehealth intervention may not be designed as stand-alone intervention. This includes training sessions and support [1]. It may be necessary to distinguish between the level of training required for the trial, and the level of training for a routine application outside of a RCT setting (discuss under item 21 – generalizability.

	1	2	3	4	5	
subitem not at all important	○	○	○	○	◉	essential

선택해제

Does your paper address subitem 5-xii? *

Copy and paste relevant sections from the manuscript (include quotes in quotation marks "like this" to indicate direct quotes from your manuscript), or elaborate on this item by providing additional information not in the ms, or briefly explain why the item is not applicable/relevant for your study

"Intervention Group
The intervention group participants underwent a 6-week self-exercise program delivered via a mobile application based self-exercise program (Figure 1). During their initial visit, participants were instructed to install the application on their mobile devices as well as received comprehensive training on its use. The application guided users through a daily 30-min self-exercise protocol, comprising stretching, strengthening and functional, and cooldown exercises (Multimedia Appendix 1). The exercise program was tailored by physiotherapists based on the patient's pain scale, functional status, and overall condition, ensuring a personalized approach to rehabilitation. To monitor the progress and adjust the intensity and content of the exercise program, the application incorporated a patient-reporting system. Before each daily exercise session, the participants quantified their pain intensity using a five-point Likert scale. After completion, they again rated their perceived exertion using a five-point Likert scale. On a weekly basis, the participants completed the KOOS–PS within the application. Accordingly, the application provided immediate visual feedback to the participants, and participants could check their weekly statistics for exercise achievement and pain levels within the application (Figure 1). Further, these data were transmitted to the supervising physiotherapist and physiatrist for remote monitoring and exercise program modifications. The exercise program was adjusted weekly to one of the three intensity levels (i.e., high, moderate, and mild) based on patient-reported outcomes (Figure 2). Owing to this approach, the application delivered a personalized self-exercise program throughout the 6-week intervention period."

6a) Completely defined pre-specified primary and secondary outcome measures, including how and when they were assessed

Does your paper address CONSORT subitem 6a? *

Copy and paste relevant sections from the manuscript (include quotes in quotation marks "like this" to indicate direct quotes from your manuscript), or elaborate on this item by providing additional information not in the ms, or briefly explain why the item is not applicable/relevant for your study

"Outcomes
The primary outcome was the total score of the Western Ontario and McMaster Universities Osteoarthritis Index (WOMAC) [26,27]. The secondary outcomes included knee pain assessed using an NRS (11-point scale), KOOS–PS [22,23], Medical Outcomes Study 36-Item Short-Form Health Survey (SF-36) [28,29], Korean version of the Geriatric Depression Scale (K-GDS) [30,31], Timed Up-and-Go (TUG) test [32], 30-second Chair Stand Test (30-CST) [33], handgrip strength test, and knee extensor strength test. Furthermore, quadriceps femoris muscle thickness was measured via ultrasonographic evaluation. The primary and secondary outcomes were evaluated at baseline and after 6 weeks. Physical function tests were assessed by an experienced physical therapist (S.A.C.), and ultrasound examinations were performed by an experienced physiatrist (J.G.D.). Adverse events were monitored throughout the study period."

6a-i) Online questionnaires: describe if they were validated for online use and apply CHERRIES items to describe how the questionnaires were designed/deployed

If outcomes were obtained through online questionnaires, describe if they were validated for online use and apply CHERRIES items to describe how the questionnaires were designed/deployed [9].

	1	2	3	4	5	
subitem not at all important	○	○	○	○	◉	essential

선택해제

Does your paper address subitem 6a-i?

Copy and paste relevant sections from manuscript text

Not applicable; we did not use online questionnaires for outcome measures

6a-ii) Describe whether and how “use” (including intensity of use/dosage) was defined/measured/monitored

Describe whether and how “use” (including intensity of use/dosage) was defined/measured/monitored (logins, logfile analysis, etc.). Use/adoption metrics are important process outcomes that should be reported in any ehealth trial.

	1	2	3	4	5	
subitem not at all important	○	○	◉	○	○	essential

선택해제

Does your paper address subitem 6a-ii?

Copy and paste relevant sections from manuscript text

"At the 6-week follow-up, participants in the intervention group completed a survey to evaluate the mobile application–guided self-exercise program. The survey collected data on self-reported exercise frequency, participants' expectations for achieving outcomes through mobile app usage, and satisfaction levels with mobile application–guided self-exercise programs, as assessed using the North American Spine Society Patient Satisfaction Index [38]."

6a-iii) Describe whether, how, and when qualitative feedback from participants was obtained

Describe whether, how, and when qualitative feedback from participants was obtained (e.g., through emails, feedback forms, interviews, focus groups).

	1	2	3	4	5	
subitem not at all important	○	○	◉	○	○	essential

선택해제

Does your paper address subitem 6a-iii?

Copy and paste relevant sections from manuscript text

"Satisfaction
At the 6-week follow-up, participants in the intervention group completed a survey to evaluate the mobile application–guided self-exercise program. The survey collected data on self-reported exercise frequency, participants' expectations for achieving outcomes through mobile app usage, and satisfaction levels with mobile application–guided self-exercise programs, as assessed using the North American Spine Society Patient Satisfaction Index [38]."

6b) Any changes to trial outcomes after the trial commenced, with reasons

Does your paper address CONSORT subitem 6b? *

Copy and paste relevant sections from the manuscript (include quotes in quotation marks "like this" to indicate direct quotes from your manuscript), or elaborate on this item by providing additional information not in the ms, or briefly explain why the item is not applicable/relevant for your study

Not applicable; there were no changes to the trial outcomes.

7a) How sample size was determined

NPT: When applicable, details of whether and how the clustering by care provides or centers was addressed

7a-i) Describe whether and how expected attrition was taken into account when calculating the sample size

Describe whether and how expected attrition was taken into account when calculating the sample size.

	1	2	3	4	5	
subitem not at all important	○	○	○	○	◉	essential

선택해제

Does your paper address subitem 7a-i?

Copy and paste relevant sections from manuscript title (include quotes in quotation marks "like this" to indicate direct quotes from your manuscript), or elaborate on this item by providing additional information not in the ms, or briefly explain why the item is not applicable/relevant for your study

"Sample Size
The sample size was calculated based on recommendations from literature on conducting pilot studies [35-37]. Assuming an expected effect size of 0.5 [8] and aiming for 90% power at a two-sided significance level of 0.05, a total of 28–30 participants (14–15 per group) were necessary for the pilot trial [36,37]. To facilitate recruitment and encourage participation, a 2:1 allocation ratio was applied, resulting in 20 and 10 participants in the intervention and control groups, respectively."

7b) When applicable, explanation of any interim analyses and stopping guidelines

Does your paper address CONSORT subitem 7b? *

Copy and paste relevant sections from the manuscript (include quotes in quotation marks "like this" to indicate direct quotes from your manuscript), or elaborate on this item by providing additional information not in the ms, or briefly explain why the item is not applicable/relevant for your study

Not applicable; we did not conduct interim analyses

8a) Method used to generate the random allocation sequence

NPT: When applicable, how care providers were allocated to each trial group

Does your paper address CONSORT subitem 8a? *

Copy and paste relevant sections from the manuscript (include quotes in quotation marks "like this" to indicate direct quotes from your manuscript), or elaborate on this item by providing additional information not in the ms, or briefly explain why the item is not applicable/relevant for your study

"Randomization
Eligible participants were randomized to the intervention or control group in a 2:1 ratio using block randomization with a block size of 6. A computer-generated randomization list was prepared at the study outset and was securely concealed in opaque envelopes. The randomization process was conducted by an independent researcher who was not involved in the outcome assessments, ensuring blinding to the treatment allocation and randomization. To further minimize bias, statistical analyses were performed by an independent analyst who was not involved in randomization process, participant allocation, or data collection. Although the study was single-blinded, with participants aware of the use of a mobile application, the study hypothesis was not disclosed to avoid potential bias in patient-reported outcomes."

8b) Type of randomisation; details of any restriction (such as blocking and block size)

Does your paper address CONSORT subitem 8b? *

Copy and paste relevant sections from the manuscript (include quotes in quotation marks "like this" to indicate direct quotes from your manuscript), or elaborate on this item by providing additional information not in the ms, or briefly explain why the item is not applicable/relevant for your study

"Randomization
Eligible participants were randomized to the intervention or control group in a 2:1 ratio using block randomization with a block size of 6. A computer-generated randomization list was prepared at the study outset and was securely concealed in opaque envelopes. The randomization process was conducted by an independent researcher who was not involved in the outcome assessments, ensuring blinding to the treatment allocation and randomization. To further minimize bias, statistical analyses were performed by an independent analyst who was not involved in randomization process, participant allocation, or data collection. Although the study was single-blinded, with participants aware of the use of a mobile application, the study hypothesis was not disclosed to avoid potential bias in patient-reported outcomes."

9) Mechanism used to implement the random allocation sequence (such as sequentially numbered containers), describing any steps taken to conceal the sequence until interventions were assigned

Does your paper address CONSORT subitem 9? *

Copy and paste relevant sections from the manuscript (include quotes in quotation marks "like this" to indicate direct quotes from your manuscript), or elaborate on this item by providing additional information not in the ms, or briefly explain why the item is not applicable/relevant for your study

"Randomization
Eligible participants were randomized to the intervention or control group in a 2:1 ratio using block randomization with a block size of 6. A computer-generated randomization list was prepared at the study outset and was securely concealed in opaque envelopes. The randomization process was conducted by an independent researcher who was not involved in the outcome assessments, ensuring blinding to the treatment allocation and randomization. To further minimize bias, statistical analyses were performed by an independent analyst who was not involved in randomization process, participant allocation, or data collection. Although the study was single-blinded, with participants aware of the use of a mobile application, the study hypothesis was not disclosed to avoid potential bias in patient-reported outcomes."

10) Who generated the random allocation sequence, who enrolled participants, and who assigned participants to interventions

Does your paper address CONSORT subitem 10? *

Copy and paste relevant sections from the manuscript (include quotes in quotation marks "like this" to indicate direct quotes from your manuscript), or elaborate on this item by providing additional information not in the ms, or briefly explain why the item is not applicable/relevant for your study

"Randomization
Eligible participants were randomized to the intervention or control group in a 2:1 ratio using block randomization with a block size of 6. A computer-generated randomization list was prepared at the study outset and was securely concealed in opaque envelopes. The randomization process was conducted by an independent researcher who was not involved in the outcome assessments, ensuring blinding to the treatment allocation and randomization. To further minimize bias, statistical analyses were performed by an independent analyst who was not involved in randomization process, participant allocation, or data collection. Although the study was single-blinded, with participants aware of the use of a mobile application, the study hypothesis was not disclosed to avoid potential bias in patient-reported outcomes."

11a) If done, who was blinded after assignment to interventions (for example, participants, care providers, those assessing outcomes) and how

NPT: Whether or not administering co-interventions were blinded to group assignment

11a-i) Specify who was blinded, and who wasn't

Specify who was blinded, and who wasn't. Usually, in web-based trials it is not possible to blind the participants [1, 3] (this should be clearly acknowledged), but it may be possible to blind outcome assessors, those doing data analysis or those administering co-interventions (if any).

	1	2	3	4	5	
subitem not at all important	○	○	○	○	◉	essential

선택해제

Does your paper address subitem 11a-i? *

Copy and paste relevant sections from the manuscript (include quotes in quotation marks "like this" to indicate direct quotes from your manuscript), or elaborate on this item by providing additional information not in the ms, or briefly explain why the item is not applicable/relevant for your study

"Randomization
Eligible participants were randomized to the intervention or control group in a 2:1 ratio using block randomization with a block size of 6. A computer-generated randomization list was prepared at the study outset and was securely concealed in opaque envelopes. The randomization process was conducted by an independent researcher who was not involved in the outcome assessments, ensuring blinding to the treatment allocation and randomization. To further minimize bias, statistical analyses were performed by an independent analyst who was not involved in randomization process, participant allocation, or data collection. Although the study was single-blinded, with participants aware of the use of a mobile application, the study hypothesis was not disclosed to avoid potential bias in patient-reported outcomes."

11a-ii) Discuss e.g., whether participants knew which intervention was the “intervention of interest” and which one was the “comparator”

Informed consent procedures (4a-ii) can create biases and certain expectations - discuss e.g., whether participants knew which intervention was the “intervention of interest” and which one was the “comparator”.

	1	2	3	4	5	
subitem not at all important	○	○	○	◉	○	essential

선택해제

Does your paper address subitem 11a-ii?

Copy and paste relevant sections from the manuscript (include quotes in quotation marks "like this" to indicate direct quotes from your manuscript), or elaborate on this item by providing additional information not in the ms, or briefly explain why the item is not applicable/relevant for your study

"Although the study was single-blinded, with participants aware of the use of a mobile application, the study hypothesis was not disclosed to avoid potential bias in patient-reported outcomes."

11b) If relevant, description of the similarity of interventions
(this item is usually not relevant for ehealth trials as it refers to similarity of a placebo or sham intervention to a active medication/intervention)

Does your paper address CONSORT subitem 11b? *

Copy and paste relevant sections from the manuscript (include quotes in quotation marks "like this" to indicate direct quotes from your manuscript), or elaborate on this item by providing additional information not in the ms, or briefly explain why the item is not applicable/relevant for your study

"Although the study was single-blinded, with participants aware of the use of a mobile application, the study hypothesis was not disclosed to avoid potential bias in patient-reported outcomes."

12a) Statistical methods used to compare groups for primary and secondary outcomes
NPT: When applicable, details of whether and how the clustering by care providers or centers was addressed

Does your paper address CONSORT subitem 12a? *

Copy and paste relevant sections from the manuscript (include quotes in quotation marks "like this" to indicate direct quotes from your manuscript), or elaborate on this item by providing additional information not in the ms, or briefly explain why the item is not applicable/relevant for your study

"Statistical Analysis
All analyses were performed using R (version 4.2.1; R Foundation for Statistical Computing, Vienna, Austria) along with a modified intention-to-treat approach. The Shapiro–Wilk test was used to assess data normality. Between-group comparisons of baseline characteristics and outcome measures at baseline and at the 6-week follow-up were performed using Fisher's exact test for categorical variables. For continuous variables, either the independent t-tests or Wilcoxon rank-sum test was used, based on the normality of the data.

The primary efficacy analysis evaluated the between-group differences in changes from baseline to 6-week follow-up. Between-group differences in changes were analyzed using independent t-tests with mean differences and 95% confidence interval (CI) when normality assumptions were met. Otherwise, the Wilcoxon rank-sum test was performed, and the median differences with 95% CI were estimated using the Hodges–Lehmann method. For within-group comparisons, paired t-test or Wilcoxon signed-rank test was performed based on the normality of the data.

Statistical significance was set at P<.05 (two-tailed), and cases with missing data were excluded from the analysis."

12a-i) Imputation techniques to deal with attrition / missing values

Imputation techniques to deal with attrition / missing values: Not all participants will use the intervention/comparator as intended and attrition is typically high in ehealth trials. Specify how participants who did not use the application or dropped out from the trial were treated in the statistical analysis (a complete case analysis is strongly discouraged, and simple imputation techniques such as LOCF may also be problematic [4]).

	1	2	3	4	5	
subitem not at all important	○	○	○	○	◉	essential

선택해제

Does your paper address subitem 12a-i? *

Copy and paste relevant sections from the manuscript (include quotes in quotation marks "like this" to indicate direct quotes from your manuscript), or elaborate on this item by providing additional information not in the ms, or briefly explain why the item is not applicable/relevant for your study

"Statistical significance was set at P<.05 (two-tailed), and cases with missing data were excluded from the analysis."

12b) Methods for additional analyses, such as subgroup analyses and adjusted analyses

Does your paper address CONSORT subitem 12b? *

Copy and paste relevant sections from the manuscript (include quotes in quotation marks "like this" to indicate direct quotes from your manuscript), or elaborate on this item by providing additional information not in the ms, or briefly explain why the item is not applicable/relevant for your study

Not applicable; we did not conduct subgroup analyses.

X26) REB/IRB Approval and Ethical Considerations [recommended as subheading under "Methods"] (not a CONSORT item)

X26-i) Comment on ethics committee approval

	1	2	3	4	5	
subitem not at all important	○	○	○	○	◉	essential

선택해제

Does your paper address subitem X26-i?

Copy and paste relevant sections from the manuscript (include quotes in quotation marks "like this" to indicate direct quotes from your manuscript), or elaborate on this item by providing additional information not in the ms, or briefly explain why the item is not applicable/relevant for your study

"This prospective, parallel, two-armed, single-blinded, single-center, randomized controlled trial was prospectively registered at ClinicalTrials.gov (NCT05197010) and approved by the Institutional Review Board of Samsung Medical Center (2021-04-004)."

x26-ii) Outline informed consent procedures

Outline informed consent procedures e.g., if consent was obtained offline or online (how? Checkbox, etc.?), and what information was provided (see 4a-ii). See [6] for some items to be included in informed consent documents.

	1	2	3	4	5	
subitem not at all important	◉	○	○	○	○	essential

선택해제

Does your paper address subitem X26-ii?

Copy and paste relevant sections from the manuscript (include quotes in quotation marks "like this" to indicate direct quotes from your manuscript), or elaborate on this item by providing additional information not in the ms, or briefly explain why the item is not applicable/relevant for your study

"Informed consent was obtained from all participants before screening."

X26-iii) Safety and security procedures

Safety and security procedures, incl. privacy considerations, and any steps taken to reduce the likelihood or detection of harm (e.g., education and training, availability of a hotline)

	1	2	3	4	5	
subitem not at all important	○	○	○	○	●	essential

선택해제

Does your paper address subitem X26-iii?

Copy and paste relevant sections from the manuscript (include quotes in quotation marks "like this" to indicate direct quotes from your manuscript), or elaborate on this item by providing additional information not in the ms, or briefly explain why the item is not applicable/relevant for your study

"Adverse events were monitored throughout the study period."

RESULTS

13a) For each group, the numbers of participants who were randomly assigned, received intended treatment, and were analysed for the primary outcome

NPT: The number of care providers or centers performing the intervention in each group and the number of patients treated by each care provider in each center

Does your paper address CONSORT subitem 13a? *

Copy and paste relevant sections from the manuscript (include quotes in quotation marks "like this" to indicate direct quotes from your manuscript), or elaborate on this item by providing additional information not in the ms, or briefly explain why the item is not applicable/relevant for your study

"Study Participants
A total of 29 participants were randomized (mean age, 70.4 ± 6.6 years; 24 [82.8%] women) with 19 and 10 allocated to the intervention and control groups, respectively. Among those in the intervention group, three did not receive the allocated intervention and were lost to follow-up. Consequently, 26 participants completed the 6-week assessment. Although some individuals were initially screened for eligibility, most simply declined participation, and the specific reasons for their refusal were not recorded. Therefore, the flow chart begins with the number of participants who consented and were randomized (Figure 3). At baseline, no significant differences in any outcome measurements were observed between the two groups, including K–L grade (Tables 1 and 2)."

13b) For each group, losses and exclusions after randomisation, together with reasons

Does your paper address CONSORT subitem 13b? (NOTE: Preferably, this is shown in a CONSORT flow diagram) *

Copy and paste relevant sections from the manuscript (include quotes in quotation marks "like this" to indicate direct quotes from your manuscript), or elaborate on this item by providing additional information not in the ms, or briefly explain why the item is not applicable/relevant for your study

"Study Participants
A total of 29 participants were randomized (mean age, 70.4 ± 6.6 years; 24 [82.8%] women) with 19 and 10 allocated to the intervention and control groups, respectively. Among those in the intervention group, three did not receive the allocated intervention and were lost to follow-up. Consequently, 26 participants completed the 6-week assessment. Although some individuals were initially screened for eligibility, most simply declined participation, and the specific reasons for their refusal were not recorded. Therefore, the flow chart begins with the number of participants who consented and were randomized (Figure 3). At baseline, no significant differences in any outcome measurements were observed between the two groups, including K–L grade (Tables 1 and 2)." "Figure 3. Study flowchart."

13b-i) Attrition diagram

Strongly recommended: An attrition diagram (e.g., proportion of participants still logging in or using the intervention/comparator in each group plotted over time, similar to a survival curve) or other figures or tables demonstrating usage/dose/engagement.

	1	2	3	4	5	
subitem not at all important	◉	○	○	○	○	essential

선택해제

Does your paper address subitem 13b-i?

Copy and paste relevant sections from the manuscript or cite the figure number if applicable (include quotes in quotation marks "like this" to indicate direct quotes from your manuscript), or elaborate on this item by providing additional information not in the ms, or briefly explain why the item is not applicable/relevant for your study

"Satisfaction
Among the 16 participants who completed the 6-week mobile application–based self-exercise program, the self-reported exercise frequency was as follows: two participants (12.5%) exercised 2–3 days per week, three participants (18.8%) exercised 3–5 days per week, five participants (31.2%) exercised more than 5 days per week, and six participants (37.5%) exercised daily (7 days per week)."

14a) Dates defining the periods of recruitment and follow-up

Does your paper address CONSORT subitem 14a? *

Copy and paste relevant sections from the manuscript (include quotes in quotation marks "like this" to indicate direct quotes from your manuscript), or elaborate on this item by providing additional information not in the ms, or briefly explain why the item is not applicable/relevant for your study

"Participants
The study participants were recruited from the musculoskeletal clinic of a tertiary academic medical center between October 2021 and December 2021."

14a-i) Indicate if critical “secular events” fell into the study period

Indicate if critical “secular events” fell into the study period, e.g., significant changes in Internet resources available or “changes in computer hardware or Internet delivery resources”

	1	2	3	4	5	
subitem not at all important	(•)	( )	( )	( )	( )	essential

선택해제

Does your paper address subitem 14a-i?

Copy and paste relevant sections from the manuscript (include quotes in quotation marks "like this" to indicate direct quotes from your manuscript), or elaborate on this item by providing additional information not in the ms, or briefly explain why the item is not applicable/relevant for your study

내 답변

14b) Why the trial ended or was stopped (early)

Does your paper address CONSORT subitem 14b? *

Copy and paste relevant sections from the manuscript (include quotes in quotation marks "like this" to indicate direct quotes from your manuscript), or elaborate on this item by providing additional information not in the ms, or briefly explain why the item is not applicable/relevant for your study

Not applicable; Our study was not stopped early.

15) A table showing baseline demographic and clinical characteristics for each group

NPT: When applicable, a description of care providers (case volume, qualification, expertise, etc.) and centers (volume) in each group

Does your paper address CONSORT subitem 15? *

Copy and paste relevant sections from the manuscript (include quotes in quotation marks "like this" to indicate direct quotes from your manuscript), or elaborate on this item by providing additional information not in the ms, or briefly explain why the item is not applicable/relevant for your study

"Table 1. Baseline characteristics of the participants." "Table 2. Outcome measures according to group at baseline."

15-i) Report demographics associated with digital divide issues

In ehealth trials it is particularly important to report demographics associated with digital divide issues, such as age, education, gender, social-economic status, computer/Internet/ehealth literacy of the participants, if known.

	1	2	3	4	5	
subitem not at all important	○	○	○	●	○	essential

선택해제

Does your paper address subitem 15-i? *

Copy and paste relevant sections from the manuscript (include quotes in quotation marks "like this" to indicate direct quotes from your manuscript), or elaborate on this item by providing additional information not in the ms, or briefly explain why the item is not applicable/relevant for your study

"Table 1. Baseline characteristics of the participants." "Visual impairment, n (%)", "Single-person household"

16) For each group, number of participants (denominator) included in each analysis and whether the analysis was by original assigned groups

16-i) Report multiple “denominators” and provide definitions

Report multiple “denominators” and provide definitions: Report N’s (and effect sizes) “across a range of study participation [and use] thresholds” [1], e.g., N exposed, N consented, N used more than x times, N used more than y weeks, N participants “used” the intervention/comparator at specific pre-defined time points of interest (in absolute and relative numbers per group). Always clearly define “use” of the intervention.

	1	2	3	4	5	
subitem not at all important	○	○	◉	○	○	essential

선택해제

Does your paper address subitem 16-i? *

Copy and paste relevant sections from the manuscript (include quotes in quotation marks "like this" to indicate direct quotes from your manuscript), or elaborate on this item by providing additional information not in the ms, or briefly explain why the item is not applicable/relevant for your study

"Among the 16 participants who completed the 6-week mobile application–based self-exercise program, the self-reported exercise frequency was as follows: two participants (12.5%) exercised 2–3 days per week, three participants (18.8%) exercised 3–5 days per week, five participants (31.2%) exercised more than 5 days per week, and six participants (37.5%) exercised daily (7 days per week). "

16-ii) Primary analysis should be intent-to-treat

Primary analysis should be intent-to-treat, secondary analyses could include comparing only “users”, with the appropriate caveats that this is no longer a randomized sample (see 18-i).

	1	2	3	4	5	
subitem not at all important	○	○	○	○	◉	essential

선택해제

Does your paper address subitem 16-ii?

Copy and paste relevant sections from the manuscript (include quotes in quotation marks "like this" to indicate direct quotes from your manuscript), or elaborate on this item by providing additional information not in the ms, or briefly explain why the item is not applicable/relevant for your study

"All analyses were performed using R (version 4.2.1; R Foundation for Statistical Computing, Vienna, Austria) along with a modified intention-to-treat approach. "

17a) For each primary and secondary outcome, results for each group, and the estimated effect size and its precision (such as 95% confidence interval)

Does your paper address CONSORT subitem 17a? *

Copy and paste relevant sections from the manuscript (include quotes in quotation marks "like this" to indicate direct quotes from your manuscript), or elaborate on this item by providing additional information not in the ms, or briefly explain why the item is not applicable/relevant for your study

"Table 4. Difference in changes in outcome measures between groups (from baseline to 6 week)" "Table 5. Changes within groups in outcome measures (from baseline to 6 week)" "Table 3. Outcome measures according to group at 6-week follow-up"

17a-i) Presentation of process outcomes such as metrics of use and intensity of use

In addition to primary/secondary (clinical) outcomes, the presentation of process outcomes such as metrics of use and intensity of use (dose, exposure) and their operational definitions is critical. This does not only refer to metrics of attrition (13-b) (often a binary variable), but also to more continuous exposure metrics such as "average session length". These must be accompanied by a technical description how a metric like a "session" is defined (e.g., timeout after idle time) [1] (report under item 6a).

	1	2	3	4	5	
subitem not at all important	○	○	○	○	◉	essential

선택해제

Does your paper address subitem 17a-i?

Copy and paste relevant sections from the manuscript (include quotes in quotation marks "like this" to indicate direct quotes from your manuscript), or elaborate on this item by providing additional information not in the ms, or briefly explain why the item is not applicable/relevant for your study

"Among the 16 participants who completed the 6-week mobile application–based self-exercise program, the self-reported exercise frequency was as follows: two participants (12.5%) exercised 2–3 days per week, three participants (18.8%) exercised 3–5 days per week, five participants (31.2%) exercised more than 5 days per week, and six participants (37.5%) exercised daily (7 days per week)."

17b) For binary outcomes, presentation of both absolute and relative effect sizes is recommended

Does your paper address CONSORT subitem 17b? *

Copy and paste relevant sections from the manuscript (include quotes in quotation marks "like this" to indicate direct quotes from your manuscript), or elaborate on this item by providing additional information not in the ms, or briefly explain why the item is not applicable/relevant for your study

Not applicable; we did not use binary outcomes, only continuous variables.

18) Results of any other analyses performed, including subgroup analyses and adjusted analyses, distinguishing pre-specified from exploratory

Does your paper address CONSORT subitem 18? *

Copy and paste relevant sections from the manuscript (include quotes in quotation marks "like this" to indicate direct quotes from your manuscript), or elaborate on this item by providing additional information not in the ms, or briefly explain why the item is not applicable/relevant for your study

Not applicable; we did not conduct a subgroup analysis.

18-i) Subgroup analysis of comparing only users

A subgroup analysis of comparing only users is not uncommon in ehealth trials, but if done, it must be stressed that this is a self-selected sample and no longer an unbiased sample from a randomized trial (see 16-iii).

	1	2	3	4	5	
subitem not at all important	○	○	◉	○	○	essential

선택해제

Does your paper address subitem 18-i?

Copy and paste relevant sections from the manuscript (include quotes in quotation marks "like this" to indicate direct quotes from your manuscript), or elaborate on this item by providing additional information not in the ms, or briefly explain why the item is not applicable/relevant for your study

Not applicable; we did not conduct a subgroup analysis.

19) All important harms or unintended effects in each group
(for specific guidance see CONSORT for harms)

Does your paper address CONSORT subitem 19? *

Copy and paste relevant sections from the manuscript (include quotes in quotation marks "like this" to indicate direct quotes from your manuscript), or elaborate on this item by providing additional information not in the ms, or briefly explain why the item is not applicable/relevant for your study

"Adverse Events
No adverse events were reported in both groups during the study period."

19-i) Include privacy breaches, technical problems

Include privacy breaches, technical problems. This does not only include physical “harm” to participants, but also incidents such as perceived or real privacy breaches [1], technical problems, and other unexpected/unintended incidents. “Unintended effects” also includes unintended positive effects [2].

	1	2	3	4	5	
subitem not at all important	○	○	◉	○	○	essential

선택해제

Does your paper address subitem 19-i?

Copy and paste relevant sections from the manuscript (include quotes in quotation marks "like this" to indicate direct quotes from your manuscript), or elaborate on this item by providing additional information not in the ms, or briefly explain why the item is not applicable/relevant for your study

"Adverse Events
No adverse events were reported in both groups during the study period."

19-ii) Include qualitative feedback from participants or observations from staff/researchers

Include qualitative feedback from participants or observations from staff/researchers, if available, on strengths and shortcomings of the application, especially if they point to unintended/unexpected effects or uses. This includes (if available) reasons for why people did or did not use the application as intended by the developers.

	1	2	3	4	5	
subitem not at all important	○	○	○	○	◉	essential

선택해제

Does your paper address subitem 19-ii?

Copy and paste relevant sections from the manuscript (include quotes in quotation marks "like this" to indicate direct quotes from your manuscript), or elaborate on this item by providing additional information not in the ms, or briefly explain why the item is not applicable/relevant for your study

"Overall, 14 participants (87.5%) of the intervention group reported scores of 1 or 2 on the North American Spine Society Patient Satisfaction Index, indicating high levels of satisfaction."

DISCUSSION

22) Interpretation consistent with results, balancing benefits and harms, and considering other relevant evidence

NPT: In addition, take into account the choice of the comparator, lack of or partial blinding, and unequal expertise of care providers or centers in each group

22-i) Restate study questions and summarize the answers suggested by the data, starting with primary outcomes and process outcomes (use)

Restate study questions and summarize the answers suggested by the data, starting with primary outcomes and process outcomes (use).

	1	2	3	4	5	
subitem not at all important	○	○	○	◉	○	essential

선택해제

Does your paper address subitem 22-i? *

Copy and paste relevant sections from the manuscript (include quotes in quotation marks "like this" to indicate direct quotes from your manuscript), or elaborate on this item by providing additional information not in the ms, or briefly explain why the item is not applicable/relevant for your study

"The findings highlight the feasibility and efficacy of a mobile application–based personalized exercise program, for elderly patients with chronic knee OA. Compared with traditional paper-based methods, the application exhibited superior outcomes in pain reduction and physical function improvement, as revealed by significant decreases in WOMAC and NRS scores. The integration of patient-reported outcomes and dynamic exercise adjustments within the application significantly contributed to maintaining high adherence rates and achieving clinically meaningful results, thereby underscoring the usefulness of digital health interventions in managing chronic musculoskeletal diseases."

22-ii) Highlight unanswered new questions, suggest future research

Highlight unanswered new questions, suggest future research.

	1	2	3	4	5	
subitem not at all important	○	○	○	○	◉	essential

선택해제

Does your paper address subitem 22-ii?

Copy and paste relevant sections from the manuscript (include quotes in quotation marks "like this" to indicate direct quotes from your manuscript), or elaborate on this item by providing additional information not in the ms, or briefly explain why the item is not applicable/relevant for your study

"Future research should focus on validating these results in large, diverse populations and investigating the long-term integration of such digital tools into routine clinical practice."

20) Trial limitations, addressing sources of potential bias, imprecision, and, if relevant, multiplicity of analyses

20-i) Typical limitations in ehealth trials

Typical limitations in ehealth trials: Participants in ehealth trials are rarely blinded. Ehealth trials often look at a multiplicity of outcomes, increasing risk for a Type I error. Discuss biases due to non-use of the intervention/usability issues, biases through informed consent procedures, unexpected events.

	1	2	3	4	5	
subitem not at all important	○	○	○	○	◉	essential

선택해제

Does your paper address subitem 20-i? *

Copy and paste relevant sections from the manuscript (include quotes in quotation marks "like this" to indicate direct quotes from your manuscript), or elaborate on this item by providing additional information not in the ms, or briefly explain why the item is not applicable/relevant for your study

"Limitations
The relatively small sample size may limit the generalizability of the findings. Additionally, the relatively short 6-week follow-up period may be insufficient to completely assess the sustainability of the observed improvements. Thus, future studies with larger sample sizes and longer follow-up periods are warranted to validate these findings and evaluate their long-term clinical impact."

21) Generalisability (external validity, applicability) of the trial findings

NPT: External validity of the trial findings according to the intervention, comparators, patients, and care providers or centers involved in the trial

21-i) Generalizability to other populations

Generalizability to other populations: In particular, discuss generalizability to a general Internet population, outside of a RCT setting, and general patient population, including applicability of the study results for other organizations

	1	2	3	4	5	
subitem not at all important	○	○	○	○	◉	essential

선택해제

Does your paper address subitem 21-i?

Copy and paste relevant sections from the manuscript (include quotes in quotation marks "like this" to indicate direct quotes from your manuscript), or elaborate on this item by providing additional information not in the ms, or briefly explain why the item is not applicable/relevant for your study

"Limitations
The relatively small sample size may limit the generalizability of the findings. Additionally, the relatively short 6-week follow-up period may be insufficient to completely assess the sustainability of the observed improvements. Thus, future studies with larger sample sizes and longer follow-up periods are warranted to validate these findings and evaluate their long-term clinical impact."

21-ii) Discuss if there were elements in the RCT that would be different in a routine application setting

Discuss if there were elements in the RCT that would be different in a routine application setting (e.g., prompts/reminders, more human involvement, training sessions or other co-interventions) and what impact the omission of these elements could have on use, adoption, or outcomes if the intervention is applied outside of a RCT setting.

	1	2	3	4	5	
subitem not at all important	◉	○	○	○	○	essential

선택해제

Does your paper address subitem 21-ii?

Copy and paste relevant sections from the manuscript (include quotes in quotation marks "like this" to indicate direct quotes from your manuscript), or elaborate on this item by providing additional information not in the ms, or briefly explain why the item is not applicable/relevant for your study

"The success of the application in maintaining adherence can be attributed to three key features: first, automated pop-up alarms delivered via participants' mobile phones are active reminders to encourage consistent participation in the exercise program. Second, the visualized statistical tracking features and weekly patient-reported symptom monitoring system of the application enable participants to continuously assess their progress, providing tangible feedback on their exercise achievements. Third, the dynamic modification of exercise intensity based on individual progression would maintain appropriate challenge levels while accommodating participants' changing capabilities. "

OTHER INFORMATION

23) Registration number and name of trial registry

Does your paper address CONSORT subitem 23? *

Copy and paste relevant sections from the manuscript (include quotes in quotation marks "like this" to indicate direct quotes from your manuscript), or elaborate on this item by providing additional information not in the ms, or briefly explain why the item is not applicable/relevant for your study

"Trial Registration: ClinicalTrials.gov NCT05197010;
https://www.clinicaltrials.gov/study/NCT05197010"

24) Where the full trial protocol can be accessed, if available

Does your paper address CONSORT subitem 24? *

Cite a Multimedia Appendix, other reference, or copy and paste relevant sections from the manuscript (include quotes in quotation marks "like this" to indicate direct quotes from your manuscript), or elaborate on this item by providing additional information not in the ms, or briefly explain why the item is not applicable/relevant for your study

"Trial Registration: ClinicalTrials.gov NCT05197010; https://www.clinicaltrials.gov/study/NCT05197010"

25) Sources of funding and other support (such as supply of drugs), role of funders

Does your paper address CONSORT subitem 25? *

Copy and paste relevant sections from the manuscript (include quotes in quotation marks "like this" to indicate direct quotes from your manuscript), or elaborate on this item by providing additional information not in the ms, or briefly explain why the item is not applicable/relevant for your study

"Acknowledgements
This work was supported by the Technology Innovation Program (20003658), funded by the Ministry of Trade, Industry & Energy (Republic of Korea). The funders had no role in the study design, collection, analysis, and interpretation of data. The authors thank all the patients for their participation."

X27) Conflicts of Interest (not a CONSORT item)

X27-i) State the relation of the study team towards the system being evaluated

In addition to the usual declaration of interests (financial or otherwise), also state the relation of the study team towards the system being evaluated, i.e., state if the authors/evaluators are distinct from or identical with the developers/sponsors of the intervention.

	1	2	3	4	5	
subitem not at all important	○	○	○	○	◉	essential

선택해제

Does your paper address subitem X27-i?

Copy and paste relevant sections from the manuscript (include quotes in quotation marks "like this" to indicate direct quotes from your manuscript), or elaborate on this item by providing additional information not in the ms, or briefly explain why the item is not applicable/relevant for your study

"Conflicts of Interest
None declared."

About the CONSORT EHEALTH checklist

As a result of using this checklist, did you make changes in your manuscript? *

- ( ) yes, major changes
- (•) yes, minor changes
- ( ) no

What were the most important changes you made as a result of using this checklist?

Level of human involvement in the METHODS section

How much time did you spend on going through the checklist INCLUDING making changes in your manuscript *

We spent approximately 2 days reviewing the checklist and making the necessary changes to our manuscript. This time included carefully going through each item on the checklist and implementing the required revisions to ensure compliance.

As a result of using this checklist, do you think your manuscript has improved? *

- (●) yes
- ( ) no
- ( ) 기타:

Would you like to become involved in the CONSORT EHEALTH group?

This would involve for example becoming involved in participating in a workshop and writing an "Explanation and Elaboration" document

- ( ) yes
- (●) no
- ( ) 기타:

선택해제

Any other comments or questions on CONSORT EHEALTH

내 답변

STOP - Save this form as PDF before you click submit

To generate a record that you filled in this form, we recommend to generate a PDF of this page (on a Mac, simply select "print" and then select "print as PDF") before you submit it.

When you submit your (revised) paper to JMIR, please upload the PDF as supplementary file.

Don't worry if some text in the textboxes is cut off, as we still have the complete information in our database. Thank you!

Final step: Click submit !

Click submit so we have your answers in our database!

제출

양식 지우기

Google Forms를 통해 비밀번호를 제출하지 마세요.

이 콘텐츠는 Google이 만들거나 승인하지 않았습니다. - 서비스 약관 - 개인정보처리방침

Does this form look suspicious? 보고서

Google 설문지
